# Cardiac Snail family of transcription factors directs systemic lipid metabolism in Drosophila

**DOI:** 10.1371/journal.pgen.1008487

**Published:** 2019-11-14

**Authors:** Ying Liu, Hong Bao, Weidong Wang, Hui-Ying Lim

**Affiliations:** 1 Department of Physiology, University of Oklahoma Health Sciences Center, Oklahoma City, Oklahoma, United States of America; 2 Department of Medicine, Section of Endocrinology, University of Oklahoma Health Sciences Center, Oklahoma City, Oklahoma, United States of America; Sanford Burnham Prebys Medical Discovery Institute, UNITED STATES

## Abstract

Maintenance of normal lipid homeostasis is crucial to heart function. On the other hand, the heart is now recognized to serve an important role in regulating systemic lipid metabolism; however, the molecular basis remains unclear. In this study, we identify the *Drosophila* Snail family of transcription factors (herein termed Sna TFs) as new mediators of the heart control of systemic lipid metabolism. Overexpression of Sna TF genes specifically in the heart promotes whole-body leanness whereas their knockdown in the heart promotes obesity. In addition, flies that are heterozygous for a *snail* deficiency chromosome also exhibit systemic obesity, and that cardiac-specific overexpression of Sna substantially reverses systemic obesity in these flies. We further show that genetically manipulating Sna TF levels in the fat body and intestine do not affect systemic lipid levels. Mechanistically, we find that flies bearing the overexpression or inhibition of Sna TFs in the postnatal heart only exhibit systemic lipid metabolic defects but not heart abnormalities. Cardiac-specific alterations of Sna TF levels also do not perturb cardiac morphology, viability, lipid metabolism or fly food intake. On the other hand, cardiac-specific manipulations of Sna TF levels alter lipogenesis and lipolysis gene expression, mitochondrial biogenesis and respiration, and lipid storage droplet 1 and 2 (Lsd-1 and Lsd-2) levels in the fat body. Together, our results reveal a novel and specific role of Sna TFs in the heart on systemic lipid homeostasis maintenance that is independent of cardiac development and function and involves the governance of triglyceride synthesis and breakdown, energy utilization, and lipid droplet dynamics in the fat body.

## Author summary

In this study, we identify a novel and specific role of the *Drosophila* Snail family of transcription factors (herein termed Sna TFs) in the heart in maintaining normal systemic lipid homeostasis. While the mammalian Snail1 transcription factor has recently been reported to regulate lipid metabolism in the mouse adipose tissue and liver, a systemic metabolic role of the Snail family factors is not known. We provide first demonstration that Sna TFs in the heart regulate lipogenesis and lipolysis, mitochondrial biogenesis and activity, and lipid droplet dynamics in the fat body. We also find that Sna TFs in the heart regulate systemic lipid levels independently of cardiac development and function, cardiac morphology and viability, and cardiac lipid metabolism. We further show that Sna TFs direct the systemic control of lipid levels from the heart and not from other metabolic tissues including the fat body and intestine. Our work therefore provides unique and striking insights into the emerging lipid metabolic functions of Sna family proteins. By defining Sna TFs as new players that mediate the heart regulation of systemic lipid homeostasis, our study also brings forward research in the field of cardiac and metabolism intersection.

## Introduction

The heart, being the sole pumping engine in the body, utilizes large amounts of fatty acids as energy-providing substrates [1]. Maintenance of cardiac lipid homeostasis is therefore important for normal heart function. On the other hand, the heart is now recognized as an important endocrine organ in the regulation of normal systemic lipid homeostasis. The notion of the heart bearing an endocrine function was first proposed based on early electron microscopic analyses of the heart atria that revealed features consistent with that of secretory cells, which include abundant rough endoplasmic reticulum, highly developed Golgi complex and electron- dense storage granules [2, 3]. Subsequently, an endocrine function of the heart on fluid and salt balance regulation was established [4, 5]. More recently, the heart has emerged as an important endocrine organ in the regulation of systemic lipid metabolism. For instance, the Mediator complex kinase subunit 13 (MED13) has been found to serve a role in the fly and mouse heart in modulating energy metabolism in a systemic manner [6–8]. Our recent work also revealed that the heart is an important source of apolipoprotein B (apoB)-containing lipoproteins (apoB- lipoproteins) and that cardiac-derived apoB-lipoproteins serve as an important determinant of whole-body lipid metabolism by controlling the uptake and transport of dietary lipids from the intestine to other tissues [9]. Together, these findings provide compelling evidence supporting a critical role of the heart in systemic lipid metabolic control; however, the underlying molecular mechanisms remain incompletely understood.

The Snail superfamily of C2H2-type zinc finger transcriptional factors is highly conserved from insects to mammals and plays a critical role in regulating epithelial to mesenchymal transition during both embryonic development and metastasis of epithelial tumors [10]. There are three snail genes in mammals (Snail1-3) and four snail genes in insects, which include *snail* (*sna*), *escargot* (*esg*) and *worniu* (*wor*) [herein termed Sna TFs]. Sna is the prototypical member of the family [11] and serves an essential role in mesoderm differentiation [12, 13]. Sna also act in a functionally redundant manner with Wor and Esg in the regulation of neuroblast development and asymmetric cell division [14–16]. Sna and Esg also exert redundant functions in the determination of wing cell fate [17]. In addition, all three Sna family proteins function redundantly and specifically in peripheral ommatidial apoptosis and patterning in the fly eye [18]. More recently, a role for Snail1 (the mouse homologue of fly Sna) [19] in the regulation of lipid metabolism has been reported. Snail1 was shown to control lipolysis and lipogenesis in the adipose tissue [20] and liver [21], respectively. Consequently, deletion of *Snail1* in the adipose tissue decreased fat mass [20], while deletion of Snail1 in the hepatocytes exacerbated, and liver-specific overexpression of Snail1 ameliorated non-alcoholic fatty liver disease in mice [21]. However, a role of Sna family proteins in the heart in governing lipid metabolism is not known.

Here we reveal that that Sna TFs act in the heart, and not in the fat body and intestine, to critically direct systemic lipid metabolism, in a manner that is independent of heart development, heart function, heart morphology, and fly food consumption. We further show that cardiac Sna TFs remotely control lipogenesis, lipolysis, mitochondrial biogenesis and function, and lipid storage droplet protein levels in the fat body. Our study therefore reveals novel and unexpected functions of Sna TFs in the heart on lipid homeostasis maintenance.

## Results

### Cardiac-specific knockdown of Sna TFs causes systemic obesity

**Fig 1:**
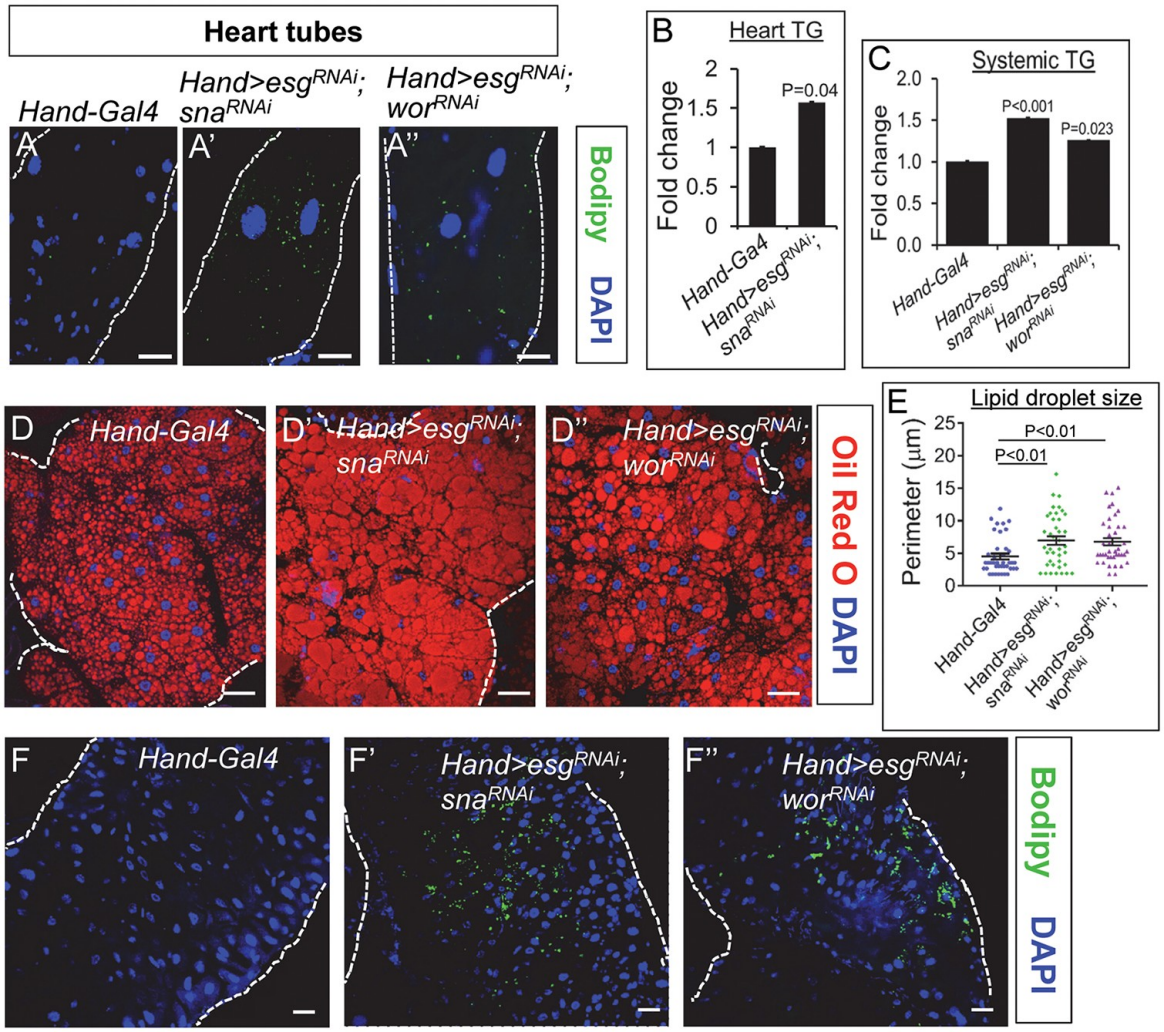
Cardiac-specific knockdown of Snail TFs induces cardiac steatosis and systemic obesity. (A-A”) Representative confocal images of hearts stained with Bodipy (green) and DAPI (blue) from 7-day old control flies (*Hand-Gal4*) (A), flies with cardiomyocyte-specific knockdown of *esg* and *sna* (*Hand>esg*^*RNAi*^; *sna*^*RNAi*^) (A’), and flies with cardiomyocyte-specific knockdown of *esg* and *wor* (*Hand>esg*^*RNAi*^; *wor*^*RNAi*^) (A”). For each genotype, 6 hearts were analyzed. Dotted lines mark the outlines of myocardial tubes. Scale bar represents 20 μm. (B) Heart TG level of 7-day old control flies (*Hand-Gal4*) and flies with cardiomyocyte-specific knockdown of *esg* and *sna* (*Hand>esg*^*RNAi*^; *sna*^*RNAi*^). TG levels (μg/μl) were normalized to total protein (μg/μl). Results are the mean ± SEM of 30–40 flies analyzed over at least 3 independent experiments and are expressed as the fold change normalized TG compared with that of the control flies (set to 1.0). (C) Whole-body TG level of 7-day old control flies (*Hand-Gal4*), and flies with cardiomyocyte-specific knockdown of Snail TF genes (*Hand>esg*^*RNAi*^; *sna*^*RNAi*^ and *Hand>esg*^*RNAi*^; *wor*^*RNAi*^). TG levels (μg/μl) were normalized to total protein (μg/μl). Results are the mean ± SEM of 30–40 flies analyzed over at least 3 independent experiments and are expressed as the fold change normalized TG compared with that of the control flies (set to 1.0). (D-D”) Representative confocal images of abdominal fat bodies stained with Oil Red O (red) and DAPI (blue) from 7-day old adult control flies (D) and flies bearing the cardiac inhibition of Sna TFs (D’-D”). For each genotype, 6 fat bodies were analyzed. Dotted lines mark the outlines of fat bodies. Scale bar represents 20 μm. (E) Lipid droplet size quantification in fat bodies from control and cardiac Sna TF-inhibited flies. 40 lipid droplets were analyzed from 6 fat bodies per genotype and results are represented as mean ± SEM. (F-F”) Representative confocal images of intestines stained with Bodipy (green) and DAPI (blue) from 7-day old adult control flies (F) and flies bearing the cardiac inhibition of Sna-TFs (F’-F”). For each genotype, 6 intestines were analyzed. Dotted lines mark the outlines of intestinal tubes. Scale bar represents 20 μm. Region 1 (R1) to R3 (hairpin region) of the *Drosophila* midgut was analyzed for Bodipy staining.

In light of the importance of lipid homeostasis in the heart, the emerged roles of Snail1 in regulating lipid metabolism in the adipose tissue and liver [20, 21] prompted us to investigate whether Sna TFs could also be involved in the regulation of lipid homeostasis in the heart, which has not been known. To address that, we first performed loss-of-function experiments by directing Sna TF knockdown in the heart using the *Hand-Gal4* driver [22]. The *Hand-Gal4* driver directs *UAS-GFP* reporter expression specifically in the cardiomyocytes (S1A, S1A’ and S1B Fig) and not in the fat body (S1C Fig) or intestine (S1D Fig), demonstrating that *Hand-Gal4* is cardiac specific. Using *Hand-Gal4* to drive the simultaneous knockdown of *esg* and *sna*, or the simultaneous knockdown of *esg* and *wor* in the heart, which led to an approximate 80%, 40%, or over 90% reduction in the cardiac levels of *sna*, *esg*, or *wor*, respectively (S2A–S2C Fig), we found an accumulation of neutral fat in the *esg*, *sna*- and esg, *wor*-double knockdown hearts, as shown by staining with Bodipy (Fig 1A and 1A”), a strong fluorescent lipophilic dye. There was also a significant increase in triglyceride (TG) level in the *esg*, *sna*-double knockdown hearts relative to control hearts (Fig 1B). Therefore, inhibition of Sna TFs specifically in the cardiomyocytes promotes cardiac steatosis.

During the process of isolating heart tissues for lipid analysis, we serendipitously observed that the abdominal fat mass appeared much denser in the cardiac-specific Sna TF-knockdown flies than in control flies (S2D and S2D’ Fig), indicating that inhibition of Sna TFs specifically in the heart could impact organismal adiposity. Intrigued by this possibility, we further investigated lipid abundance in the cardiac Sna TF-inhibited flies and found significantly increased TG levels in the whole body (Fig 1C), fat body (S2E Fig) and intestine (S2F Fig), relative to control flies. Neutral fat staining using another lipophilic fluorescent dye Oil Red O revealed stronger lipid accumulation (Fig 1D-1D”), and significantly greater lipid droplet size (Fig 1E) in the fat body of the cardiac-specific Sna TF-knockdown flies compared to control flies. In addition to the fat body, the fly midgut has emerged as an important metabolic tissue involved in peripheral body fat storage and it also serves as the major site of dietary lipid absorption [23]. Moreover, the fly midgut, which normally accumulates very little fat, exhibits significantly increased fat deposition in response to high dietary fat intake [9], suggesting that it can serve as a sensitive system in determining changes in systemic lipid homeostasis. We therefore assessed ectopic lipid accumulation in the intestine using Bodipy staining, and our results showed the presence of ectopic lipid deposition in the intestine of the cardiac-specific Sna TF-knockdown flies but not in control flies (Fig 1F-1F”). As adiposity is an important determinant of insulin resistance [24, 25], we next assessed whether the fat body of the cardiac-specific Sna-knockdown flies, which stores more fat than the control fat body, could have altered insulin sensitivity. Indeed, in response to insulin stimulation, Akt phosphorylation, a molecular indicator of activated insulin signaling, was significantly reduced in the fat body of the cardiac *esg*, *sna*-knockdown flies compared to that in control flies (S2G and S2G’ Fig). Together, these data provide first evidence supporting a novel role of Sna TFs in the heart on the regulation of systemic lipid metabolism.

### Cardiac-specific overexpression of Sna TFs promotes whole body leanness

To further investigate our unexpected discovery that Sna TFs in the heart control systemic lipid metabolism, we next performed gain-of-function experiments by directing *sna*, *wor*, or *esg* overexpression specifically in the cardiomyocytes using *Hand-Gal4*, which is expected to drive Sna TF overexpression throughout the entire myocardial tube (see S1A’ Fig). To confirm the overexpression of Sna TFs in the cardiomyocytes, we examined the levels of Sna TFs in the Sna-, Esg-, and Wor-overexpressing hearts. While control cardiomyocytes displayed low abundances of all three Sna TFs (S3A, S3B and S3C Fig), overexpression of Sna, Esg, or Wor using *Hand-Gal4* heightened Sna, Esg, or Wor amounts, respectively, in the cardiomyocytes with predominantly cytoplasmic and occasional nuclear (arrows) accumulation of Sna and Esg (S3A’ and S3B’ Fig) and predominantly nuclear accumulation of Wor (arrows, S3C’ Fig). Notably, we found no obvious increase in the level of Sna TFs in the fat body of the cardiac Sna TF-overexpressing flies compared to control flies (S3D–S3F’ Fig), thus confirming the cardiac specificity of *Hand-Gal4* (S1C Fig) and also demonstrating that overexpression of Sna TFs in the heart does not non-autonomously induce Sna TFs in the fat body.

**Fig 2:**
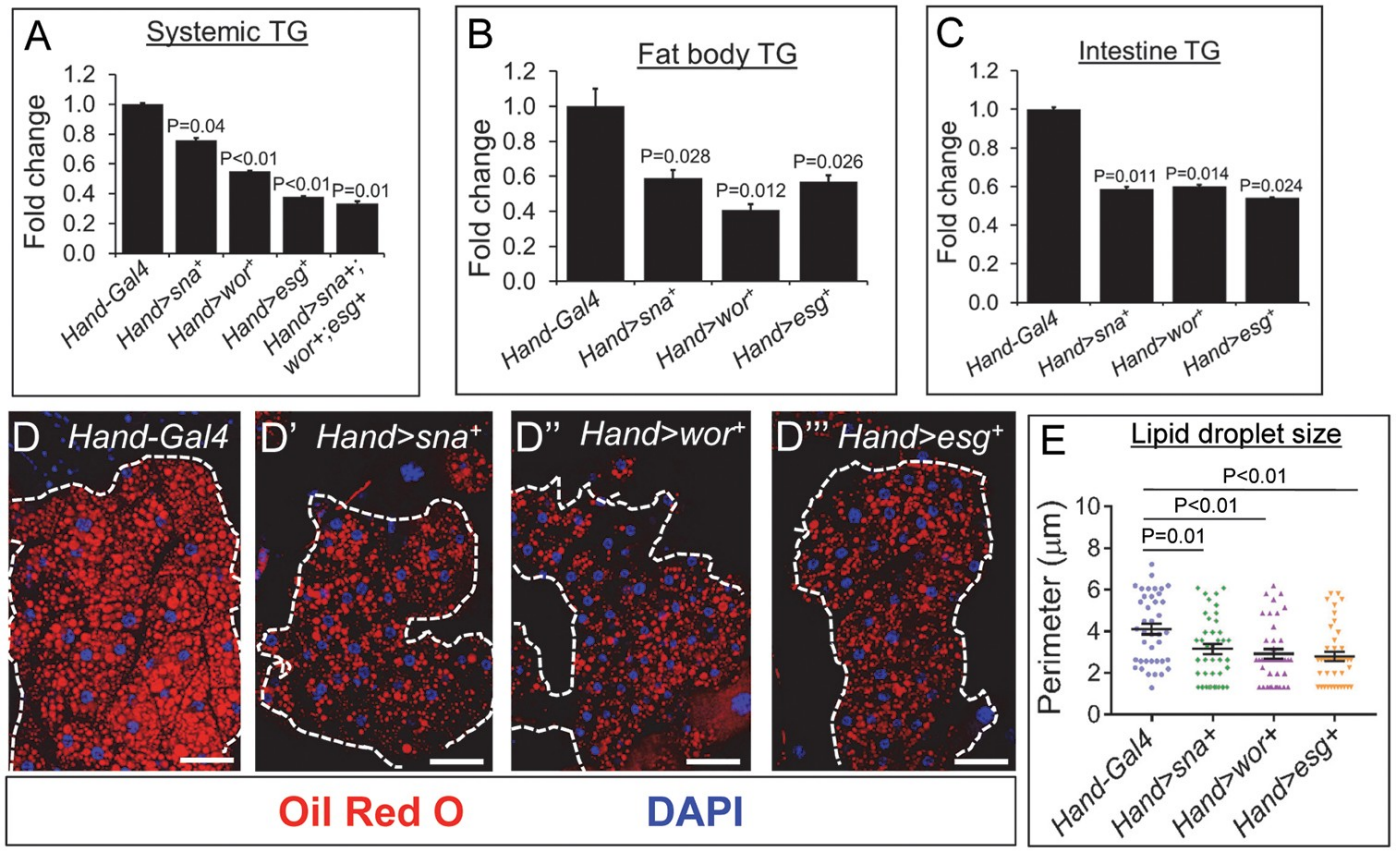
Cardiac-specific overexpression of Snail TFs promotes systemic leanness. (A-C) Whole-body TG level (A), fat body TG level (B), and intestine TG level (C) of 7-day old control flies (*Hand-Gal4*), and flies with cardiomyocyte-specific single overexpression of Sna TFs (*Hand>sna*^+^, *Hand>wor*^+^ or *Hand>esg*^+^) or triple overexpression of Sna TFs (*Hand>sna*^+^; *wor*^+^; *esg*^+^). TG levels (μg/μl) were normalized to total protein (μg/μl). Results are the mean ± SEM of 30–40 flies analyzed over at least 3 independent experiments and are expressed as the fold change normalized TG compared with that of the control flies (set to 1.0). (D-D”‘) Representative confocal images of abdominal fat bodies stained with Oil Red O (red) and DAPI (blue) from 7-day old adult control (D) and cardiac Sna-TF overexpressing flies (D’- D”‘). For each genotype, 6 fat bodies were analyzed. Dotted lines mark the outlines of fat bodies. Scale bar represents 20 μm. (E) Lipid droplet size quantification in fat bodies from control and cardiac Sna-TF overexpressing flies. 40 lipid droplets were analyzed from 6 fat bodies per genotype and results are represented as mean ± SEM.

Our further analyses of systemic lipid content in the cardiac Sna TF-overexpressing flies revealed significant decreases in whole-body (Fig 2A), fat body (Fig 2B), and intestinal (Fig 2C) TG levels in these flies relative to control flies. Neutral fat staining of the fat body showed an obvious reduction in lipid storage in the cardiac Sna-TF overexpressing flies compared to control flies (Fig 2D-2D”‘). There was also a significant decrease in fat body lipid droplet size in the cardiac Sna-TF overexpressing flies compared to control flies (Fig 2E). Further indicative of reduced adiposity, the abdominal fat mass in the cardiac Sna TF- overexpressing flies was sparser than in control flies, with the presence of multiple gaps (arrows, S4A-S4A”‘ Fig). We also detected significantly enhanced Akt phosphorylation in the fat body of the cardiac Sna-overexpressing flies compared to that in control flies (S4B and S4B’ Fig). Previous studies have found that insulin upregulates Snail1 in the adipose tissue and liver [20, 21]. As the fat bodies isolated from flies have been transiently exposed to insulin before assaying for Akt phosphorylation (S4B and S4B’ Fig), it is possible that insulin stimulates Sna expression differentially in fat bodies between the cardiac Sna-overexpressing and Sna TF- knockdown flies which can then impinge on Akt phosphorylation. To rule out this possibility, we first determined whether insulin induces Sna expression in the fat body, and we found that to be the case (S4E and S4E’ Fig). However, the extent of Sna induction by insulin was comparable among the fat bodies of control flies, cardiac Sna-overexpressing flies, and cardiac Sna TF-knockdown flies (S4E’, S4E”‘ and S4F Fig), indicating that insulin-stimulated Sna induction does not contribute to the differential fat body insulin sensitivity seen in the cardiac Sna TF-manipulated flies (S2G, S2G’, S4B and S4B’ Figs). In addition, using the *tGPH* transgenic fly line, which has been used as an in situ indicator of PI3K activity (another molecular indicator of activated insulin signaling) [26], we observed a stronger membrane-associated tGFP signal in the fat body of the cardiac Sna-overexpressing flies compared to in control flies (S4C-S4D’ Fig), thus substantiating our findings on phospho-Akt and together strengthening the notion of enhanced fat body insulin sensitivity in the cardiac Sna-overexpressing flies. In all, our results indicate that overexpression of Sna TFs specifically in the cardiomyocytes promotes the development of obesity and insulin resistance, lipid metabolic phenotypes that are opposite to those seen with the inhibition of Sna TFs in the heart (Fig 1).

### Systemic obesity in the Sna TF-deficient flies was substantially rescued upon the cardiac expression of Sna

**Fig 3:**
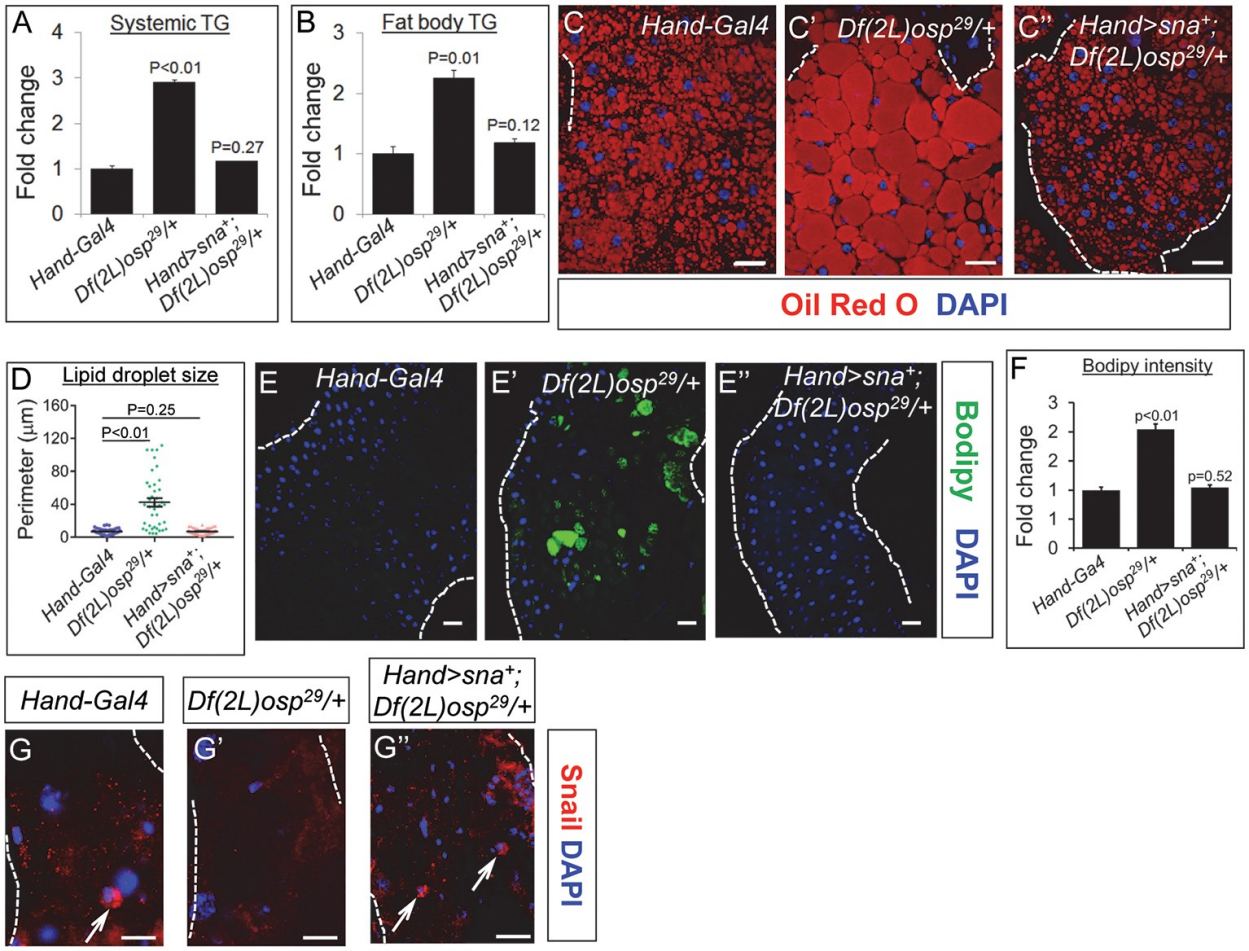
Cardiac expression of Snail substantially reverses the systemic obesity in Snail TF-deficient flies. (A-B) Whole-body TG level (A) and fat body TG level (B) of 7-day old control flies (*Hand-Gal4*), flies heterozygous for *Df(2L)osp*^*29*^ (*Df(2L)osp*^*29*^*/+*), and *Df(2L)osp*^*29*^ heterozygous flies bearing cardiac-specific expression of Sna (*Df(2L)osp*^*29*^*/+;Hand>sna*^+^). TG levels (μg/μl) were normalized to total protein (μg/μl). Results are the mean ± SEM of 30–40 flies analyzed over at least 3 independent experiments and are expressed as the fold change normalized TG compared with that of the control flies (set to 1.0). (C-C”) Representative confocal images of abdominal fat bodies stained with Oil Red O (red) and DAPI (blue) from 7-day old control flies (*Hand-Gal4*) (C), flies heterozygous for *Df(2L)osp*^*29*^ (*Df(2L)osp*^*29*^*/+*) (C’), and *Df(2L)osp*^*29*^ heterozygous flies bearing cardiac-specific expression of Sna (*Df(2L)osp*^*29*^*/+; Hand>sna*^+^) (C”). For each genotype, 6 fat bodies were analyzed. Dotted lines mark the outlines of fat bodies. Scale bar represents 20 μm. (D) Lipid droplet size quantification in fat bodies from control flies, flies heterozygous for *Df(2L)osp*^*29*^ (*Df(2L)osp*^*29*^*/+*), and *Df(2L)osp*^*29*^ heterozygous flies bearing cardiac-specific expression of Sna (*Df(2L)osp*^*29*^*/+; Hand>sna*^+^). 40 lipid droplets were analyzed from 6 fat bodies per genotype and results are represented as mean ± SEM. (E-E”) Representative confocal images of intestines stained with Bodipy (green) and DAPI (blue) from 7-day old control flies (*Hand-Gal4*) (E), flies heterozygous for *Df(2L)Osp*^*29*^ (*Df(2L)Osp*^*29*^*/+*) (E’), and *Df(2L)Osp*^*29*^ heterozygous flies bearing cardiac-specific expression of Sna (*Df(2L)Osp*^*29*^*/+; Hand>sna*^+^) (E”). For each genotype, 6 intestines were analyzed. Dotted lines mark the outlines of intestinal tubes. Scale bar represents 20 μm. Region 1 (R1) to R3 (hairpin region) of the *Drosophila* midgut was analyzed for Bodipy staining. (F) Bodipy fluorescence quantification in intestines from control flies, flies heterozygous for *Df(2L)osp*^*29*^ (*Df(2L)osp*^*29*^*/+*), and *Df(2L)osp*^*29*^ heterozygous flies bearing cardiac-specific expression of Sna (*Df(2L)osp*^*29*^*/+; Hand>sna*^+^). Three separate areas were analyzed from each of 6 intestines per genotype and results are represented as mean ± SEM. (G-G”) Representative confocal images of Sna (red) immunostaining and DAPI (blue) co- staining in fixed heart preparations from 1-wk-old control flies (*Hand-Gal4*) (G), flies heterozygous for *Df(2L)osp*^*29*^ (*Df(2L)osp*^*29*^ */+*) (G’), and *Df(2L)osp*^*29*^ heterozygotes bearing cardiac-specific expression of Sna (*Df(2L)osp*^*29*^*/+; Hand>sna*^+^) (G”). Dotted lines outline the myocardial heart tube. Scale bar represents 20 μm. Arrows indicate nuclear localization of Sna in cardiomyocytes.

To further corroborate the notion that Sna TFs serve an important role in the heart on the governance of systemic lipid metabolism, we asked whether flies that are heterozygous for a deficiency chromosome that uncovers many genes including *sna*, *wor*, and *esg* (herein termed *Df(2L)osp29/+*) would also exhibit systemic obese phenotypes and that replenishing Sna level only in the heart of these flies would be sufficient to rescue their systemic lipid metabolic deficits. Indeed, we observed that the *Df(2L)osp29/+* flies display systemic obese phenotypes which are reminiscent of the cardiac inhibition of Sna TFs (Fig 1), including significant increases in systemic and fat body TG levels (Fig 3A and 3B), dramatic increase in the deposition of neutral lipids in the fat body (Fig 3C and 3C’), significantly enlarged lipid droplet size (Fig 3D), and ectopic accumulation of neutral lipids in the intestine (Fig 3E, 3E’ and 3F). Next, we targeted the expression of Sna in the hearts of the *Df(2L)osp29/+* flies and detected a restoration of normal Sna level in the *Df(2L)osp29/+* hearts (Fig 3G-3G”). We further found that there was a corresponding correction of systemic lipid metabolic defects in the *Df(2L)osp29/+* flies by the cardiac restoration of Sna level. As shown in Fig 3A and 3B, the excessive amounts of TG in the whole body and fat body of the *Df(2L)osp29/+* flies were significantly brought down to levels insignificant to that of control levels upon concomitant overexpression of Sna in the heart. Furthermore, the robust accumulation of neutral fat in the fat body of the *Df(2L)osp29/+* flies (Fig 3C’) was suppressed to a level comparable to that in control flies by the cardiac-specific reintroduction of Sna (Fig 3C and 3C”). The expanded lipid droplet size in the *Df(2L)osp29/+* fat body adipocytes was also corrected to normal level with the concurrent expression of Sna in the heart (Fig 3D). Likewise in the intestine, the ectopic deposition of neutral fat in the *Df(2L)osp29/+* intestine (Fig 3E’) was significantly suppressed to a level similar to that of control flies by the cardiac-targeted reintroduction of Sna (Fig 3E, 3E” and 3F). Hence, restoring Sna level only in the heart is sufficient to substantially alleviate the elevated systemic TG level and excess fat body lipid accumulation phenotypes in the *Df(2L)osp29/+* flies.

### Genetic alterations of Sna TFs in fat body or intestine do not perturb systemic TG levels

To further determine whether the unexpected role of Sna TFs in regulating systemic lipid metabolism is cardiac specific or whether it also occurs in other metabolic tissues including the fat body and intestine, we examined the effects of overexpressing or knocking down Sna TFs specifically in the fat body or intestine on systemic lipid levels, by utilizing two fat body-specific Gal4 drivers, *pumpless* (*Ppl)-Gal4* and *R4-Gal4* [9] and two intestinal-specific *Gal4* drivers, *caudal* (*Cd*)*-Gal4* and *Myo1A-Gal4* drivers [27]. Our results showed that Sna overexpression or Sna TF inhibition in the fat body (S5A and S5B Fig) or intestine (S5C and S5D Fig) did not elicit any significant effects on systemic TG levels. Moreover, overexpression of Sna or knockdown of Sna TFs in the fat body did not alter lipid abundance in the heart (S5E-S5E” Fig). In contrast, fat body-specific overexpression of Sna or knockdown of Sna TFs strongly decreased or increased, respectively, lipid accumulation in the fat body (S5F-S5F” Fig). Together, these observations support a cardiac-specific function of Sna TFs on systemic lipid homeostasis maintenance.

### Cardiac Sna TFs regulate systemic lipid metabolism in a manner that is independent of heart development

So far, our results point toward a central and specific function of Sna TFs in the heart on systemic lipid metabolism. This prompted us to investigate the underlying mechanisms. We first determined the possibility that the overexpression or knockdown of Sna TFs in the heart could disrupt normal heart development which may then give rise to the systemic lipid metabolic phenotypes. This is especially relevant in light of our evoking of Sna TF level changes in the heart using *Hand-Gal4*, which is active during the developmental stage [22]. We further reason that the genetic manipulations of Sna TF levels in the heart only during the postnatal stage will allow us to exclude the confounding effects of heart development in our analysis of the cardiac Sna TF-regulation of systemic lipid metabolism. To genetically manipulate Sna TFs in the postnatal heart only, we utilized the inducible Gal80ts/*Gal4* system [28]. At the non-permissive temperature (17°C), Gal80ts binds Gal4 to inhibit the *Gal4*-mediated transcriptional activation while at the permissive temperature (29°C), Gal80ts dissociates from Gal4 to permit the *Gal4*- mediated gene expression. To ascertain that *Hand-Gal4* was effectively suppressed during the non-permissive stage, we performed heart immunostainings in newly-eclosed flies that have been reared from embryonic stage to adult eclosion at 17°C. We found that control flies (*Tub- Gal80*^*ts*^; *Hand-Gal4*) and *Tubulin-Gal80*^*ts*^, *Hand>sna*^+^ flies exhibited comparably low levels of Sna in their hearts (S6A and S6A’ Fig), indicating that the suppression of *Hand-Gal4* by *Tubulin-Gal80*^*ts*^ is complete throughout development. Next, to determine that suppression of *Hand-Gal4* by *Tubulin-Gal80*^*ts*^ is reversed during the adult stage, newly-eclosed control and *Tubulin- Gal80*^*ts*^, *Hand>sna*^+^ flies that have been reared continuously at 17°C were transferred to 29°C for 7 days, followed by their heart immunostainings for Sna. We found that compared to control flies, the *Tubulin-Gal80*^*ts*^; *Hand>sna*^+^ flies now displayed detectably increased cardiac level of Sna (S6B and S6B’ Fig).

**Fig 4:**
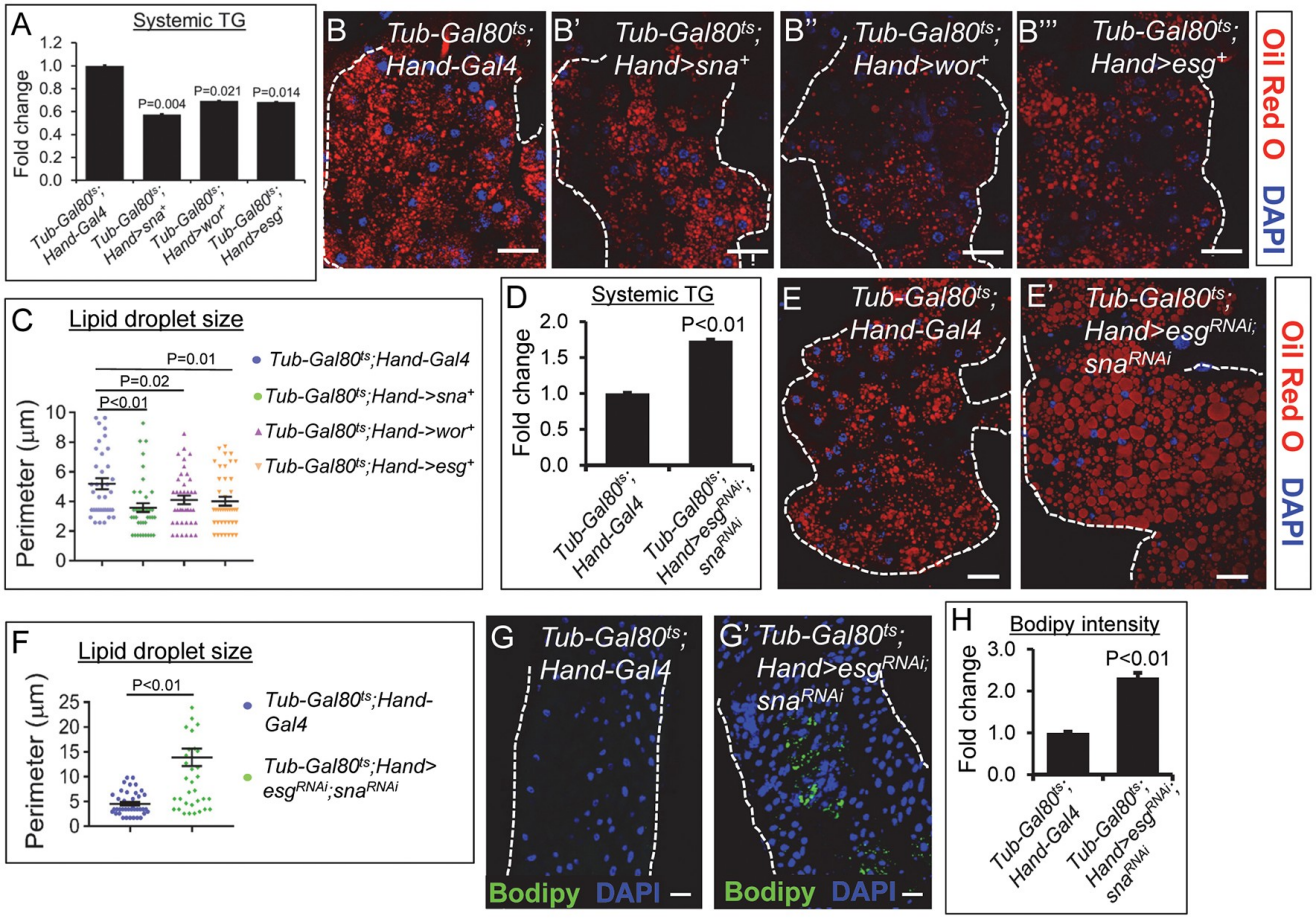
Overexpression or inhibition of Snail TFs in post-natal hearts only promotes overall leanness or obesity, respectively. (A) Whole-body TG level of 3-week old control flies (*Tub-Gal80*^*ts*^; *Hand-Gal4*) and flies that overexpress Sna TFs in postnatal hearts only (*Tub-Gal80*^*ts*^; *Hand>sna*^+^ or *Tub-Gal80*^*ts*^; *Hand>wor*^+^ or *Tub-Gal80*^*ts*^; *Hand>esg*^+^). TG levels (μg/μl) were normalized to total protein (μg/μl). Results are the mean ± SEM of 30–40 flies analyzed over at least 3 independent experiments and are expressed as the fold change normalized TG compared with that of the control flies (set to 1.0). (B-B”‘) Representative confocal images of abdominal fat bodies stained with Oil Red O (red) and DAPI (blue) from 3-wk-old control flies (B) and flies that overexpress Sna TFs in postnatal hearts only (B’-B”‘). For each genotype, 6 fat bodies were analyzed. Dotted lines mark the outlines of fat bodies. Scale bar represents 20 μm. (C) Lipid droplet size quantification in fat bodies from control flies and flies that overexpress Sna TFs in postnatal hearts only. 40 lipid droplets were analyzed from 6 fat bodies per genotype and results are represented as mean ± SEM. (D) Whole-body TG level of 7-day old control flies (*Tub-Gal80*^*ts*^; *Hand-Gal4*) and flies with SnaTF gene knockdowns in postnatal hearts only (*Tub-Gal80*^*ts*^; *Hand>esg*^*RNAi*^, sna^*RNAi*^ or *Tub- Gal80*^*ts*^; *Hand>esg*^*RNAi*^, wor^*RNAi*^). TG levels (μg/μl) were normalized to total protein (μg/μl). Results are the mean ± SEM of 30–40 flies analyzed over at least 3 independent experiments and are expressed as the fold change normalized TG compared with that of the control flies (set to 1.0). (E-E”) Representative confocal images of abdominal fat bodies stained with Oil Red O (red) and DAPI (blue) from 7-day-old control flies (E) and flies with Sna TF gene knockdowns (E’) in postnatal hearts only. For each genotype, 6 fat bodies were analyzed. Dotted lines mark the outlines of fat bodies. Scale bar represents 20 μm. (F) Lipid droplet size quantification in fat bodies from control flies and flies with Sna TF gene knockdowns in postnatal hearts only. 40 lipid droplets were analyzed from 6 fat bodies per genotype and results are represented as mean ± SEM. (G-G’) Representative confocal images of intestines stained with Bodipy (green) and DAPI (blue) from 7-day-old control flies (G) and flies with Sna TF gene knockdowns (G’) in postnatal hearts only. For each genotype, 6 intestines were analyzed. Dotted lines mark the outlines of intestinal tubes. Scale bar represents 20 μm. Region 1 (R1) to R3 (hairpin region) of the *Drosophila* midgut was analyzed for Bodipy staining. (H) Bodipy fluorescence quantification in intestines from control flies and flies with Sna TF gene knockdowns in postnatal hearts only. Three separate areas were analyzed from each of 6 intestines per genotype and results are represented as mean ± SEM.

Having confirmed that the temperature shift effectively induces *Hand-Gal4*-mediated Sna overexpression during the postnatal stage only, we then analyzed the associated lipid metabolic effects. Compared to control flies, flies with the overexpression of Sna TFs in postnatal heart only exhibited significantly decreased systemic TG content (Fig 4A) and reduced neutral fat accumulation in the fat body (Fig 4B-4B”‘). The fat body lipid droplet sizes in these flies were significantly lower than in control flies (Fig 4C). Intestinal TG levels were significantly decreased as well in these flies compared to control flies (S6C Fig). Consistent with their display of lean phenotypes, these flies also harbored reduced quantities of fat mass in the abdomen, as reflected by the presence of more and larger gaps within their fat mass (arrows, S6D’-S6D”‘ Fig) compared to the control abdomens (S6D Fig).

Next, we determined whether inhibition of Sna TFs in the heart only during the postnatal stage could adversely affect systemic lipid homeostasis. For this purpose, we generated progenies bearing *Tubulin-Gal80*^*ts*^, *Hand-Gal4*, and *UAS-sna*^*RNAi*^ and *UAS-esg*^*RNAi*^ or *UAS- esg*^*RNAi*^ and *UAS-wor*^*RNAi*^ and reared them from embryonic stage to adult eclosion at 17°C followed by a temperature shift of the newly-eclosed adults to 29°C. After 7–10 days of their adulthood at 29°C, the flies were subjected to systemic lipid metabolic analyses. We observed significantly higher levels of systemic TG in the adult-only, cardiac-specific *esg*, *sna*-double knockdown flies and *esg*, *wor*-double knockdown flies compared to control flies (Fig 4D). Lipid deposition and lipid droplet size were significantly higher in the fat body (Fig 4E, 4E’ and 4F) of these flies compared to control flies. Ectopic lipid deposition was detected in the midgut of these flies compared to control flies as well (Fig 4G, 4G’ and 4H). Finally, inhibition of Sna TFs in the postnatal heart only was associated with a thicker fat mass in the abdomen compared to the control (S6E and S6E’ Fig). As our findings showed that genetically altering Sna TF levels only in the adult heart evoked systemic lipid metabolic phenotypes similar to and to the same extent as those caused by the cardiac manipulations of Sna TFs throughout lifespan (Figs 1 and 2), we conclude that cardiac Sna TFs direct systemic lipid homeostasis in a manner independent of heart development.

### Alterations of cardiac Sna TF levels do not perturb adult heart morphology, adult heart function, cardiac lipid metabolism gene expressions, or fly food consumption

**Fig 5:**
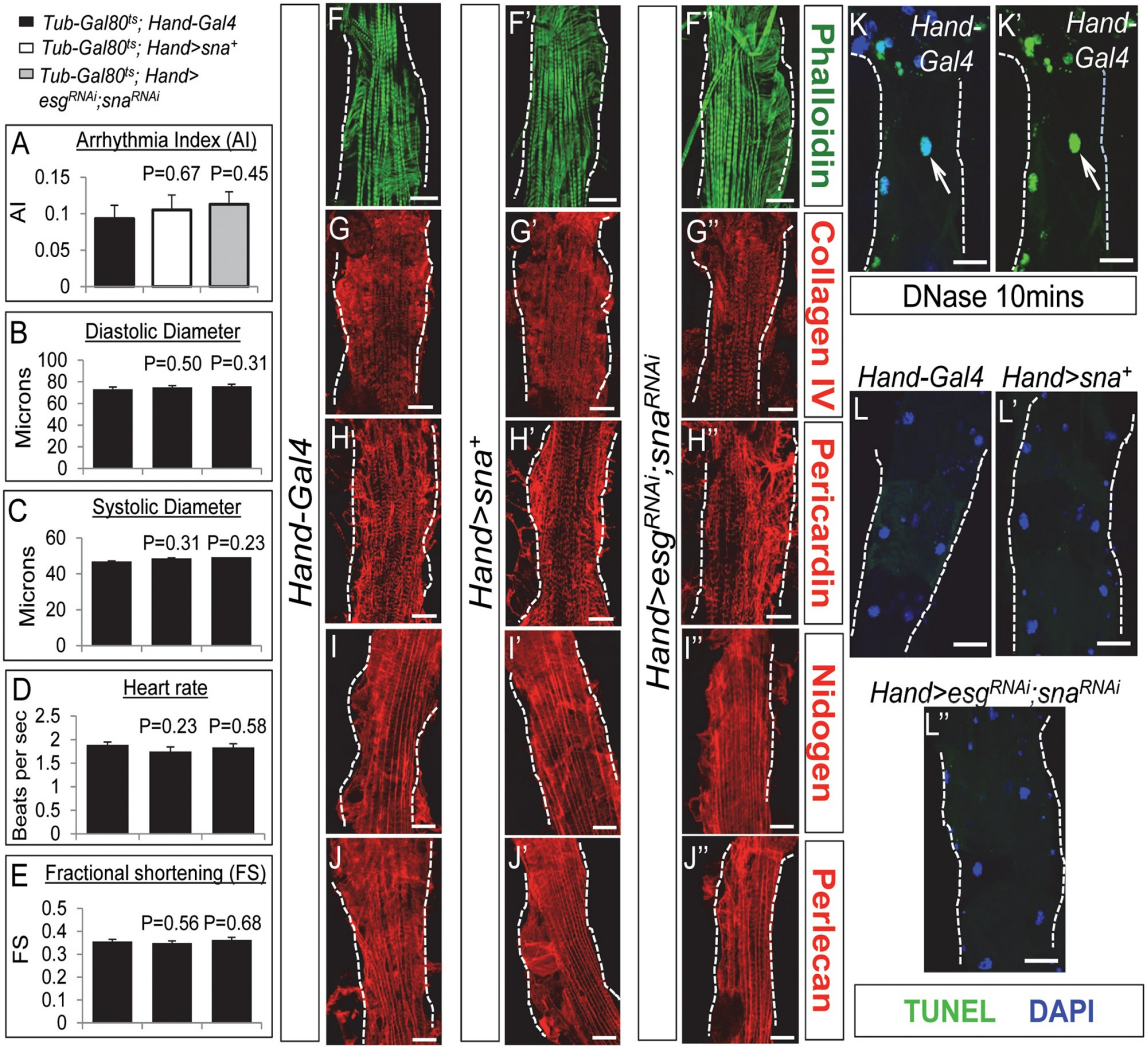
Adult heart morphology and function are not perturbed by the cardiac-specific overexpression or inhibition of Snail TFs. (A-E) Arrhythmia index (A), diastolic diameter (B), systolic diameter (C), heart rate (D), and fractional shortening (E) of 7-day-old control flies (*Tub-Gal80*^*ts*^; *Hand-Gal4*), flies that overexpress Sna in postnatal hearts only (*Tub-Gal80*^*ts*^; *Hand>sna*^+^), and flies with inhibition of Sna TFs in postnatal hearts only (*Tub-Gal80*^*ts*^; *Hand>esg*^*RNAi*^, sna^*RNAi*^). Results are the mean ± SEM of 25–30 flies per genotype. (F-F”) Representative confocal images of 7-day-old hearts stained by phalloidin for actin filaments (green) in control flies (*Hand-Gal4*) (F), flies with cardiomyocyte-specific overexpression of Sna (*Hand>sna*^+^) (F’), and flies with cardiomyocyte-specific knockdown of Sna TF genes (*Hand>esg*^*RNAi*^; *sna*^*RNAi*^) (F”). For each genotype, 6 hearts were analyzed. Dotted lines mark the outlines of heart tubes. Scale bar represents 20 μm. (G-G”) Representative confocal images of 7-day-old hearts immunostained for the ECM core component Collagen IV (red) in control flies (*Hand-Gal4*) (G), flies with cardiomyocyte-specific overexpression of Sna (*Hand>sna*^+^) (G’), and flies with cardiomyocyte-specific knockdown of Sna TF genes (*Hand>esg*^*RNAi*^; *sna*^*RNAi*^) (G”). For each genotype, 6 hearts were analyzed. Dotted lines mark the outlines of heart tubes. Scale bar represents 20 μm. (H-H”) Representative confocal images of 7-day-old hearts immunostained for the ECM-binding protein Pericardin (red) in control flies (*Hand-Gal4*) (H), flies with cardiomyocyte-specific overexpression of Sna (*Hand>sna*^+^) (H’), and flies with cardiomyocyte-specific knockdown of Sna TF genes (*Hand>esg*^*RNAi*^; *sna*^*RNAi*^) (H”). For each genotype, 6 hearts were analyzed. Dotted lines mark the outlines of heart tubes. Scale bar represents 20 μm. (I-I”) Representative confocal images of 7-day-old hearts immunostained for the ECM core component Nidogen (red) in control flies (*Hand-Gal4*) (I), flies with cardiomyocyte-specific overexpression of Sna (*Hand>sna*^+^) (I’), and flies with cardiomyocyte-specific knockdown of Snail TF genes (*Hand>esg*^*RNAi*^; *sna*^*RNAi*^) (I”). For each genotype, 6 hearts were analyzed. Dotted lines mark the outlines of heart tubes. Scale bar represents 20 μm. (J-J”) Representative confocal images of 7-day-old hearts immunostained for the ECM core component Perlecan (red) in control flies (*Hand-Gal4*) (J), flies with cardiomyocyte-specific overexpression of Sna (*Hand>sna*^+^) (J’), and flies with cardiomyocyte-specific knockdown of Snail TF genes (*Hand>esg*^*RNAi*^; *sna*^*RNAi*^) (J”). For each genotype, 6 hearts were analyzed. Dotted lines mark the outlines of heart tubes. Scale bar represents 20 μm. (K-L”) TUNEL assay for the detection of apoptosis in 7-day-old hearts of control flies (*Hand- Gal4*) treated with DNase (K-K’), and hearts not treated with DNase from control flies (L), cardiac Sna-overexpressing flies (*Hand>sna*^+^) (L’), and flies with cardiomyocyte-specific knockdown of Snail TF genes (*Hand>esg*^*RNAi*^; *sna*^*RNAi*^) (L”). In K-K’, apoptotic cardiomyocytes are shown with green fluorescence in the nuclei (white arrows) that have been stained with DAPI (blue). Dotted lines mark the outlines of heart tubes. Scale bar represents 20 μm.

Our results above provided evidence supporting a role of Sna TFs in the adult heart on the maintenance of systemic lipid metabolism; however, it is still possible that alterations of Sna TF levels in the postnatal heart only could affect adult heart performance that in turn leads to systemic lipid metabolic aberrations. For instance, the Sna TF-induced adult cardiac dysfunction can disrupt hemolymph circulation and consequently nutrient distribution to other tissues, thereby evoking systemic effects on lipid metabolism. To assess this possibility, we analyzed the contractility of hearts with the overexpression or knockdown of Sna TFs only during the postnatal stage. Although such manipulations of Sna TF levels were sufficient to evoke systemic lipid metabolic defects (Fig 4), there were no significant impairments on heart function, as we found no obvious abnormalities in heartbeat regularity (Fig 5A), diastolic dimension (Fig 5B), systolic dimension (Fig 5C), heart rate (Fig 5D), and fractional shortening (Fig 5E) compared to that in control flies. Given that heart function and heart morphology are intimately linked, we further determined whether cardiac morphology could be impacted by alterations of Sna TF levels in the heart. First, we examined the organization of the myofibrils in the Sna- overexpressing hearts and the *esg*, *sna*-double knockdown hearts, by performing phalloidin staining of the actin filaments. We observed no detectable differences in myofibril arrangements between these hearts and the control hearts (Fig 5F-5F”). Next, given the known role of Snail’s involvement in tissue fibrosis [29–32], we assessed fibrosis and remodeling in the Snail- transgenic or knockdown hearts by examining the abundance of extracellular matrix (ECM) components, which are indicators of cardiac fibrosis [33]. Our results showed that the amounts of ECM proteins Collagen IV (Fig 5G-5G”), Pericardin (Fig 5H-5H”), Nidogen (Fig 5I-5I”), and Perlecan (Fig 5J-5J”) were comparable between the Sna-overexpressing hearts or the *esg*, *sna*-double silenced hearts and control hearts. We also assessed whether manipulations of Sna TF levels in the heart would cause cardiomyocyte death, by performing the TUNEL (terminal deoxynucleotidyl transferase dUTP nick end labeling) assay. Control hearts treated with DNase I showed the presence of fluorescent labeling in their nuclei (arrows, Fig 5K and 5K’), indicating the occurrence of apoptosis as expected. In the absence of DNase treatment, TUNEL assay detected no increased nuclear fluorescent label in the Sna- overexpressing or Sna TF-knockdown hearts compared to control hearts (Fig 5L-5L”), indicating that cardiomyocyte Sna TF manipulations do not evoke cardiac apoptosis.

Next, to determine whether cardiac-specific alterations in Sna TF levels could affect cardiac lipid metabolism, we probed the transcript levels of several genes involved in free fatty acid (FFA) biosynthesis [*fatty acid synthase* (*Fas*)], TG lipogenesis [*acylglycerol phosphate acyltransferase* (*AGPAT-3*) and *Lipin*], lipolysis [*Brummer* (*Bmm*)], mitochondrial biogenesis (*PGC-1*/*spargel*), and lipid droplet physiology [*Lipid surface droplet-2*(*Lsd-2*)]. Our results showed that expressions of these genes were comparable between the *esg*, *sna*-double knockdown hearts and control hearts (S7A–S7F Fig), arguing against the notion that alterations in Sna TF levels in the heart perturb cardiac lipid homeostasis. Finally, we investigated whether food intake could be affected in flies with altered levels of Sna TF in the heart as that could impact the initial supply of nutrients to cause an imbalance between energy intake and energy expenditure and ultimately disrupt normal systemic lipid homeostasis. For that, we measured food intake in adult flies using the capillary feeder (CAFÉ) assay [34]. Our results showed that both the cardiac- specific Sna TF-overexpressing and the *esg*, *wor*- and *esg*, *sna*-double knockdown flies exhibited comparable food consumption as the control flies (S7G and S7H Fig), indicating that fly feeding behavior is not perturbed by the cardiac genetic manipulations of Sna TF levels. We conclude that changing Sna TF levels in the heart do not compromise cardiac function, integrity, viability, lipid metabolism, and fly food consumption.

### Cardiac Sna TFs control lipogenesis and lipolysis in the fat body

**Fig 6:**
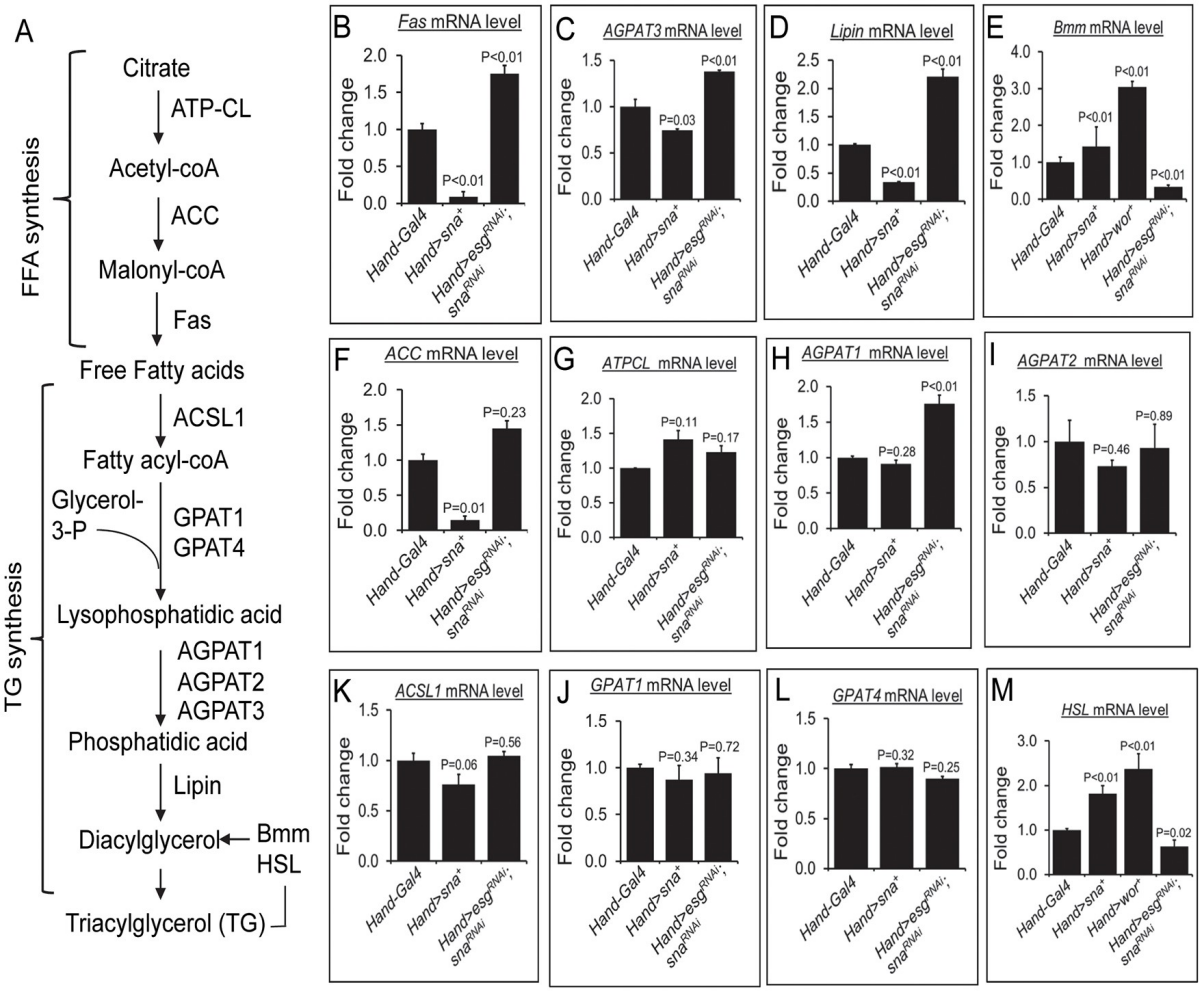
Fat body lipogenic and lipolytic gene expressions in flies with cardiac-specific overexpression or knockdown of Snail TFs. (A) Schematic diagram of lipogenesis and lipolytic pathways. FFA, free fatty acid; TG, triglyceride. (B-M) Relative mRNA level of FFA synthesis genes *Fas* (B), *ACC* (F), and *ATPCL* (G), TG lipogenesis genes *AGPAT3* (C), *Lipin* (D), *AGPAT1* (H), *AGPAT2* (I), *ACSL1* (K), *GPAT1* (J), and *GPAT4* (L), and TG lipolytic genes *Bmm* (E) and *HSL* (M) in adult fat bodies of control flies (*Hand-Gal4*), flies with cardiomyocyte-specific overexpression of Sna (*Hand>sna*^+^), and flies with cardiomyocyte-specific knockdown of Sna TF genes (*Hand>esg*^*RNAi*^; *sna*^*RNAi*^). Results are the mean ± SEM of 30–40 fat bodies analyzed over 3 independent experiments and are expressed as the fold change compared with control fat bodies (set to 1.0).

In adipocytes, lipid droplet size reflects a balance of TG synthesis (lipogenesis) and hydrolysis (lipolysis) [35]. We therefore determined whether lipogenesis or lipolysis could be changed in the fat body of flies with the cardiac gain- or loss-of-function of Sna TFs. We first examined the fat body expression of a few genes involved in lipogenesis and lipolysis, including *Fas*, *AGPAT-3*, *Lipin*, and *Bmm* (Fig 6A). We found that the transcript levels of *Fas*, *AGPAT-3*, and *Lipin* were significantly decreased in the fat body of the cardiac Sna-overexpressing flies and increased in the fat body of the cardiac Sna TF-inhibited flies, compared to control flies (Fig 6B–6D). Conversely, the transcript level of *Bmm* was significantly increased or decreased, respectively, in the Sna TF-overexpressing or knockdown flies compared to control flies (Fig 6E). Therefore, in contrast to their seemingly lack of regulation by Sna TFs in the heart (S7A–S7D Fig), *Fas*, *AGPAT-3*, *Lipin*, and *Bmm* in the fat body appear to be regulated by Sna TFs in the heart, albeit indirectly as heart-specific changes in Sna TFs do not induce Sna TFs in the fat body (S3D–S3F’ Fig). Intrigued by these observations, we interrogated the expression of several more lipogenesis and lipolytic genes in the fat body upon the manipulations of Sna TFs in the heart. We found that the fat body transcript levels of the FFA biosynthesis genes *acyl-coA carboxylase* (*ACC*) but not *ATP-citrate lyase* (*ATPCL*) was significantly reduced in the cardiac Sna-overexpressing flies and elevated in the cardiac Sna TF-knockdown flies relative to control flies (Fig 6F and 6G). In addition, the fat body transcript level of the TG lipogenesis gene *AGPAT-1* was also significantly higher in the cardiac Sna TF-knockdown flies relative to control flies (Fig 6H), whereas *AGPAT2* transcription in the fat body remained unchanged by the cardiac alterations of Sna TFs (Fig 6I). In addition to *AGPAT1*-*3* and *Lipin*, which are all involved in the later steps of the TG biosynthesis process (Fig 6A), we also examined *acyl-coA synthetase 1* (*ACSL1*) and *glycerol-3-phosphate acyltransferase-1* and -4 (*GPAT1* and *GPAT4*), which are TG biosynthesis genes that participate in the earlier steps of TG synthesis (Fig 6A). We found no obvious difference in the fat body transcript levels of these genes between the cardiac Sna- overexpressing flies or cardiac Sna TF-knockdown flies and control flies (Fig 6K-6L). Last but not least, we found that the lipolytic gene *Hormone-sensitive lipase* (*HSL*) in the fat body was significantly up-regulated in the cardiac Sna TF-overexpressing flies and significantly down- regulated in the cardiac Sna TF-knockdown flies (Fig 6M), similar to that observed for *Bmm* (Fig 6E). Taken together, our findings indicate that lipogenesis and lipolysis in the fat body are controlled by Sna TFs in the heart.

### Cardiac Sna TFs control mitochondrial biogenesis and function in the fat body

**Fig 7:**
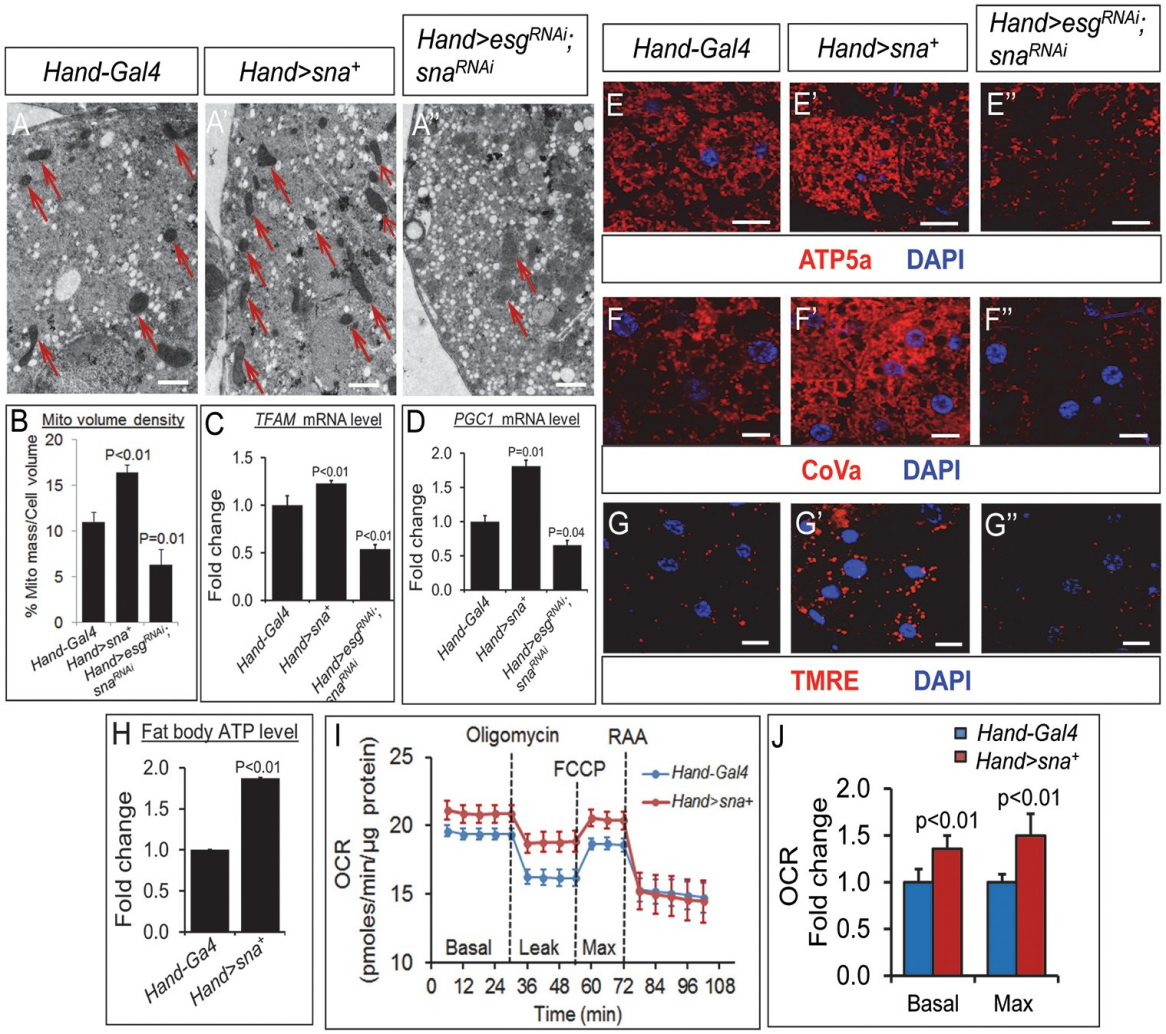
Mitochondrial biogenesis and function in the fat body are perturbed by cardiac- specific manipulations of Snail TF levels. (A-A”) Representative images of transmission electron micrographs of the fat body of control flies (A), flies with cardiomyocyte-specific overexpression of Sna (*Hand>sna*^+^) (A’), and flies with cardiomyocyte-specific knockdown of Snail TF genes (*Hand>esg*^*RNAi*^; *sna*^*RNAi*^) (A”) showing mitochondria as electron dense structures (red arrows). Magnification = 2500X. Scale bar = 1 μm. (B) Quantification of mitochondrial volume density (% of cell volume occupied by mitochondria) in the fat body of control flies, flies with cardiomyocyte-specific overexpression of Sna (*Hand>sna*^+^), and flies with cardiomyocyte-specific knockdown of Snail TF genes (*Hand>esg*^*RNAi*^; *sna*^*RNAi*^). Fat bodies from 10 adult flies were analyzed per genotype. (C-D) Relative mRNA level of mitochondrial biogenesis genes *TFAM* (C) and *PGC-1*/*spargel* (D) in fat bodies of control flies (*Hand-Gal4*), flies with cardiomyocyte-specific overexpression of Sna (*Hand>sna*^+^), and flies with cardiomyocyte-specific knockdown of Sna TF genes (*Hand>esg*^*RNAi*^; *sna*^*RNAi*^). Results are the mean ± SEM of 60 fat bodies analyzed over 3 independent experiments and are expressed as the fold change compared with control fat bodies (set to 1.0). (E-E”) Representative confocal images of abdominal fat bodies immunostained for the mitochondrial ATP synthase subunit ATP5A (red) and co-stained with DAPI (blue) from 7-day- old control flies (*Hand-Gal4*) (E), flies with cardiomyocyte-specific overexpression of Sna (*Hand>sna*^+^) (E’), and flies with cardiomyocyte-specific knockdown of Snail TF genes (*Hand>esg*^*RNAi*^; *sna*^*RNAi*^) (E”). For each genotype, 6 fat bodies were analyzed. Scale bar represents 20 μm. (F-F”) Representative confocal images of abdominal fat bodies immunostained for the mitochondrial Cytochrome c oxidase subunit 5A/CoVa (red) and co-stained with DAPI (blue) from 7-day-old control flies (*Hand-Gal4*) (F), flies with cardiomyocyte-specific overexpression of Sna (*Hand>sna*^+^) (F’), and flies with cardiomyocyte-specific knockdown of Snail TF genes (*Hand>esg*^*RNAi*^; *sna*^*RNAi*^) (F”). For each genotype, 6 fat bodies were analyzed. Scale bar represents 20 μm. (G-G”) Representative confocal images of abdominal fat bodies stained with the TMRE dye for mitochondrial membrane potential detection (red) and DAPI (blue) from 7-day-old control flies (*Hand-Gal4*) (G), flies with cardiomyocyte-specific overexpression of Sna (*Hand>sna*^+^) (G’), and flies with cardiomyocyte-specific knockdown of Snail TF genes (*Hand>esg*^*RNAi*^; *sna*^*RNAi*^) (G”). For each genotype, 6 fat bodies were analyzed. Scale bar represents 20 μm. (H) Steady-state ATP level in the fat bodies of 7-day-old control flies (*Hand-Gal4*), flies with cardiomyocyte-specific overexpression of Sna (*Hand>sna*^+^), and flies with cardiomyocyte- specific knockdown of Snail TF genes (*Hand>esg*^*RNAi*^; *sna*^*RNAi*^). Results are the mean ± SEM of fat bodies isolated from 30–40 flies analyzed over 3 independent experiments and are expressed as the fold change compared with control fat bodies (set to 1.0). (I) Oxygen consumption rate in fat bodies from 3–4 day-old control flies (*Hand-Gal4*) and flies with cardiomyocyte-specific overexpression of Sna (*Hand>sna*^+^). Fat bodies from 15 adult flies were used per well. Oligomycin (final 10μM), FCCP (final 0.4μM), Rotenone (final 3μM) and Antimycin (final 12μM) were added at the indicated time point. Data points are represented as mean ± SEM. (J) Basal and Maximal (Max) rate of respiration in fat bodies from 3–4 day-old control flies (*Hand-Gal4*) and flies with cardiomyocyte-specific overexpression of Sna (*Hand>sna*^+^).

Adipocyte lipogenesis and lipolysis are closely linked to energy expenditure [36–39]. We therefore assessed energy expenditure in the fat body of the cardiac Sna TF-manipulated flies, by evaluating mitochondrial mass and respiration, which are the classical means of assessing energy expenditure [40, 41]. Mitochondrial mass, which represents the net balance between mitochondrial biogenesis and degradation, in the fat body was evaluated by several means. First, transmission electron microscopy (TEM) was used to determine mitochondrial biogenesis. In the fat body of the cardiac Sna-overexpressing flies, there were greater numbers of mitochondria (red arrows, Fig 7A and 7A’) and significantly increased mitochondrial volume density (per unit area occupied by mitochondria) (Fig 7B) compared to control flies. In contrast, the fat body of the cardiac-specific Sna TF-knockdown flies displayed fewer numbers of mitochondria (red arrows, Fig 7A and 7A”) and significantly decreased mitochondrial volume density relative to control flies (Fig 7B). Mitochondria in the fat body of the cardiac- specific Sna TF-knockdown flies also appeared less electron dense than those in control flies (red arrows, Fig 7A and 7A”). Second, real-time PCR analysis of the transcript levels of mitochondrial biogenesis genes *dTFAM* and *PGC-1*/*spargel* revealed their significant up-regulation in the fat body of the cardiac Sna-overexpressing flies and their significant down-regulation in the fat body of the cardiac Sna TF-knockdown flies, compared to control flies (Fig 7C and 7D). Third, immunostainings were performed for the mitochondrial markers ATP5A and Cytochrome c Oxidase Subunit Va (CoVa), which are subunits of the mitochondrial ATP synthase and electron transport chain complex IV, respectively. In addition, the mitochondrial-specific fluorescent dye tetra-methylrhodamine ester TMRE was also employed to assess mitochondria with normal membrane potential. In all three cases, we detected stronger immunosignals for ATP5a and CoVa and higher fluorescence level for TMRE in the fat body of the cardiac-specific Sna transgenic flies compared to control flies (Fig 7E, 7E’, 7F, 7F’, 7G and 7G’). Conversely, ATP5a and CoVa immunosignals and TMRE fluorescence levels were reduced in the fat body of flies bearing the cardiac-specific knockdown of *sna* and *esg* relative to control flies (Fig 7E, 7E”, 7F, 7F”, 7G and 7G”). As a corollary of an increase in mitochondrial biogenesis, we detected an increase in mitochondrial activity in the fat body of the cardiac-specific Sna TF-overexpressing flies relative to control flies, as shown by the significantly increased ATP production (Fig 7H) and basal and maximal mitochondrial oxygen consumption rate (OCR) (Fig 7I and 7J). No significant difference in ATP production in the fat body was found between the cardiac-specific Sna TF-inhibited flies and control flies (S7I Fig), which could be due to compensatory mechanisms that produce more ATP when mitochondrial function is inhibited [42]. Moreover, we found that expression of several genes involved in the mitochondrial fatty acid β-oxidation pathway, including those encoding acyl-coA dehydrogenases (CG17544 and CG9527) and acyl-coA oxidase 57D-p (CG9707), were significantly induced in the fat bodies of the cardiac Sna-overexpressing flies (S8A–S8C Fig), indicating that mitochondrial function is also elevated in the fat tissues of these flies. In sum, we conclude that energy expenditure is altered in the fat body of flies with the cardiac gain- or loss-of-function of Sna TFs.

### Fat body Lsd1 and Lsd2 levels are altered upon the genetic increase or decrease in cardiac Sna TF levels

**Fig 8:**
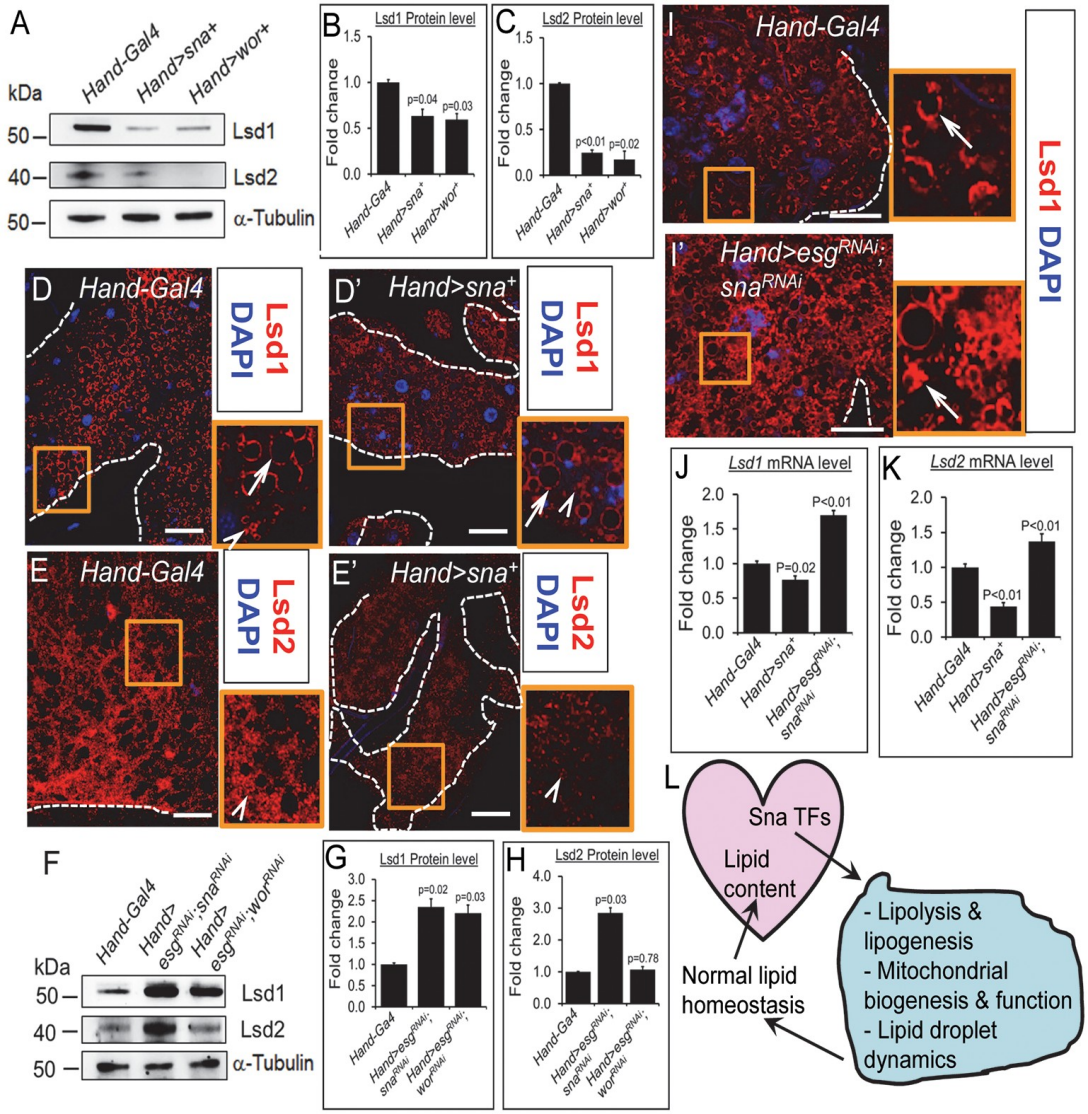
Alterations in fat body Lsd1 and Lsd2 levels upon the genetic increase or decrease in cardiac Snail TF levels. (A) Western blot analyses of Lsd1 and Lsd2 in the fat bodies of 1-week old control flies (*Hand- Gal4*), flies with cardiomyocyte-specific overexpression of Sna (*Hand>sna*^+^) or Wor (*Hand>wor*^+^). α-Tubulin was used as loading control. About thirty μg of protein was loaded per lane. (B-C) Quantification of the Lsd1 (B) and Lsd2 (C) bands from Western blot experiments, as shown in (A). Results were normalized to α-tubulin levels and expressed as fold change compared to control fat bodies. Data are represented as mean ± SEM. (D-E’) Representative confocal images of abdominal fat bodies immunostained for the Lsd1 (red) and DAPI (blue) from 7-day-old control flies (*Hand-Gal4*) (D) and flies with cardiomyocyte- specific overexpression of Sna (*Hand>sna*^+^) (D’) and for Lsd2 (red) and DAPI (blue) from 7-day- old control flies (*Hand-Gal4*) (E) and flies with cardiomyocyte-specific overexpression of Sna (*Hand>sna*^+)^ (E’). For each genotype, 6 fat bodies were analyzed. Dotted lines mark the outlines of fat bodies. Scale bar represents 20 μm. In all cases, a representative portion of the fat body (orange box) is shown in an accompanying magnified (40X) blowout box. Arrows and arrowheads in blowout box denote Lsd1 or Lsd2 localization on large (arrows) and small (arrowheads) lipid droplets, respectively, in the fat body. (F) Western blot analyses of Lsd1 and Lsd2 in the fat bodies of 1-week old control flies (*Hand- Gal4*) and flies with cardiomyocyte-specific inhibition of Sna TFs (*Hand>esg*^*RNAi*^; *sna*^*RNAi*^ or *Hand>esg*^*RNAi*^; *wor*^*RNAi*^). α-Tubulin was used as loading control. About thirty μg of protein was loaded per lane. (G-H) Quantification of the Lsd1 (G) and Lsd2 (H) bands from Western blot experiments, as shown in (F). Results were normalized to α-tubulin levels and expressed as fold change compared to control fat bodies. Data are represented as mean ± SEM. (I-I’) Representative confocal images of abdominal fat bodies immunostained for the Lsd1 (red) and DAPI (blue) from 7-day-old control flies (*Hand-Gal4*) (I) and flies with cardiomyocyte- specific inhibition of Sna TFs (*Hand>esg*^*RNAi*^; *sna*^*RNAi*^) (I’). For each genotype, 6 fat bodies were analyzed. Dotted lines mark the outlines of fat bodies. Scale bar represents 20 μm. In I and I’, a representative portion of the fat body (orange box) is shown in an accompanying magnified (40X) blowout box. Arrows and arrowheads in blowout box denote Lsd1 or Lsd2 localization on large (arrow) and small (arrowhead) lipid droplets, respectively, in the fat body. (J-K) Relative mRNA level of *Lsd1* (J) and *Lsd2* (K) in adult fat bodies of control flies (*Hand- Gal4*), flies with cardiomyocyte-specific overexpression of Sna (*Hand>sna*^+^), and flies with cardiomyocyte-specific knockdown of Sna TF genes (*Hand>esg*^*RNAi*^; *sna*^*RNAi*^). Results are the mean ± SEM of 60 hearts analyzed over 3 independent experiments and are expressed as the fold change compared with control fat bodies (set to 1.0). (L) Summary diagram depicting Sna TFs in the heart (pink) directing various lipid metabolic responses in the fat body (blue), which culminate in the maintenance of normal systemic lipid homeostasis that in turn is important for regulating normal cardiac lipid content.

In mammals, the perilipin family of lipid droplet-associated proteins plays a crucial role in restricting adipocyte lipolysis under basal condition [43], and both the mammalian perilipins and their *Drosophila* homologues, lipid storage droplet 1 and 2 (Lsd1 and Lsd2), are important modulators of organismal fat content [44–48]. We therefore examined whether Lsd1 and Lsd2 levels in the fat body could be altered upon the genetic gain or loss of Sna TFs in the heart. As shown by Western blot analyses, both Lsd1 and Lsd2 protein abundance were significantly diminished in the fat body of the cardiac-specific Sna- or Wor-overexpressing flies relative to control flies (Fig 8A–8C). In addition to Western blot analysis, we also performed immunostainings of Lsd1 and Lsd2 in the fat body. Previous studies have shown that Lsd1 is localized to most lipid droplets whereas Lsd2 is mainly localized on the surfaces of small lipid droplets in the larval fat body [47]. Similarly, in mammals, Perilipin1 (mammalian homologue of fly Lsd1) are localized to large lipid droplets while ADRP/Perilipin2 (mammalian homologue of fly Lsd2) localizes to small lipid droplets [49]. Consistent with these findings on Lsd1 and Lsd2 lipid droplet localizations, we detected Lsd1 immunosignal on both large and small lipid droplets and Lsd2 immunosignal mainly on the small lipid droplets in the adult fat body of control flies (Fig 8D, 8E and 8I). Immunostaining of Lsd1 or Lsd2 in the fat body of the cardiac-specific Sna-overexpressing flies showed their detectable reductions on lipid droplet surfaces compared to that in control flies (Fig 8D’ and 8E’, compare to Fig 8D and 8E respectively). Therefore, the lowering of systemic fat content is associated with diminution of Lsd protein quantities in the fat body in these flies. For the reciprocal experiments, we analyzed Lsd protein concentrations in the fat body of flies bearing the cardiac-specific silencing of *esg* and *sna*, or *esg* and *wor*, flies which display enhanced systemic lipid levels (Fig 1). Examination of their fat body Lsd protein abundances using Western blot analyses (Fig 8F–8H) or immunostaining (Fig 8I and 8I’) revealed significantly heightened amounts of Lsd1 and Lsd2 than in control flies, thus corroborating a positive correlation between fat body Lsd protein levels and whole-body TG content in these flies. In agreement with the pattern of fat body Lsd1 and Lsd2 protein level changes in response to altered Sna TF levels in the heart, we found that the fat body Lsd1 and Lsd2 transcript levels were significantly reduced in the cardiac Sna-overexpressing flies and significantly increased in the cardiac Sna TF-inhibited flies, relative to control flies (Fig 8J and 8K).

To further probe whether changing Lsd level in the fat body would directly alter systemic lipid homeostasis, and importantly whether these alterations phenocopy those seen with the cardiac manipulations of Sna TFs, we genetically overexpressed or knocked down Lsd genes specifically in the fat body followed by TG level and fat body lipid storage analyses. We observed that relative to control flies, flies in which *lsd1* or *lsd2* has been silenced in the fat body exhibited significantly lower whole-body TG concentration (S9A Fig). Moreover, the *lsd2*- silenced fat body displayed significantly decreased fat body TG level (S9B Fig) and lipid storage (S9C and S9C’ Fig) compared to control fat body. In reciprocal experiments, overexpression of Lsd2 but not Lsd1 in the fat body led to significantly heightened systemic TG level compared to control flies (S9D Fig) [44]. Notably, the co-overexpression of Lsd2 and Lsd1 specifically in the fat body synergistically elevated systemic TG level (S9D Fig). Likewise, co-overexpression of Lsd1 and Lsd2 in the fat body led to significantly enhanced TG level (S9E Fig) and increased fat accumulation (S9F and S9F’ Fig) in the fat body compared to control fat body. These findings document that loss or gain of Lsd1 and Lsd2 in the fat body phenocopied the cardiac-specific gain or loss of Sna TFs (Figs 1 and 2), respectively. The functional correlations between fat body Lsd proteins and cardiac Sna TFs are also in agreement with the expression data showing that cardiac Sna TF overexpression diminishes Lsd1 and Lsd2 abundance in the fat body whereas cardiac Sna TF inhibition heightens Lsd1 abundance in the fat body (Fig 8). In all, these results provide a strong causal link between cardiac Sna TFs and fat body Lsd proteins on systemic lipid metabolic regulation.

## Discussion

In this study, we reveal a novel role of the Sna TFs as critical mediators of the heart control of systemic lipid metabolism. We show that siRNA-mediated knockdown of Sna TFs specifically in the heart evokes cardiac steatosis. Interestingly, systemic TG levels and lipid accumulation in the extra-cardiac tissues were also heightened in association with the heart- specific inhibition of Sna TFs. The systemic lipid effects were confirmed by our further demonstrations that cardiac-specific overexpression of Sna TFs lowers whole-body TG levels and lipid accumulation in the fat body. Moreover, the replenishment of Sna level only in the heart is sufficient to fully reverse systemic obesity in the Sna TF-deficient flies. Inhibition or overexpression of Sna TFs specifically in fat body or intestine does not elicit any detectable changes in systemic lipid content, which correlate with those reported in the adipose- and hepatic-specific *Snail1*-knockout mice in which adipose and hepatic fat content are altered, respectively, but systemic (plasma, skeletal muscle, and kidney) TG levels remain unperturbed [20, 21]. Together, these data indicate a central role of Sna TFs in the heart and not in other metabolic tissues in directing systemic lipid homeostasis.

In probing the mechanisms that underlie the cardiac Sna TF-governance of systemic lipid metabolism, we determined that the genetic increase or decrease in Sna TF levels in the heart only during the postnatal stage promotes systemic leanness or obesity, respectively, but do not affect adult heart performance, thus ruling out any heart developmental and functional effects on the cardiac Sna TF-regulation of systemic lipid metabolism. We also found that the genetic increase or decrease in cardiac Sna TF levels does not appear to disrupt cardiac myofibrillar organization nor causes fibrosis or apoptosis in the heart, thus ruling out any heart morphology or viability effects on the cardiac Sna TF-governance of systemic lipid metabolism. Our results further showed that inhibition of Sna TFs in the heart does not affect cardiac lipid metabolism gene expression, thereby arguing against a cardiac-autonomous role of Sna TFs in controlling lipid metabolism which when disrupted, could give rise to cardiac steatosis seen in the Sna TF-inhibited heart. Instead, we propose that steatosis in the Sna TF-inhibited hearts is an indirect consequence of systemic lipid homeostasis alterations caused by the cardiac inhibition of Sna TF levels (Fig 8L). Finally, we showed that flies that bear the cardiac overexpression or knockdown of Sna TFs exhibit food intake that is comparable to control flies, thus excluding the contribution of food consumption in the cardiac Sna TF-regulation of systemic lipid metabolism.

On the other hand, we have observed that lipid metabolism gene expressions in the fat body are changed in association with alterations in cardiac Sna TFs. For example, in the fat body of cardiac Sna-overexpressing flies, genes involved in *de novo* TG lipogenesis are down- regulated, in particular genes that encode AGPAT3 and Lipin which catalyze the later steps of the TG synthesis pathway (Fig 6). Interestingly, while GPAT has been considered to be the rate limiting enzyme in the TG lipogenesis pathway [50], genetic defects in AGPAT and Lipin have led to the suggestion that these later-acting enzymes may also be rate-limiting [51]. In contrast, genes involved in TG lipolysis are up-regulated in the fat body of cardiac Sna-overexpressing flies, which include the rate-limiting gene *bmm* encoding the *Drosophila* homologue of mammalian ATGL. Reciprocally, in the cardiac Sna TF-knockdown flies, genes involved in *de novo* TG lipogenesis are up-regulated while genes involved in TG lipolysis are down-regulated in the fat body. The fat body of the cardiac Sna TF-overexpressing or knockdown flies also display altered energy utilization, as evident by changes in mitochondrial mass and function. Notably, we found an increase in mitochondrial biogenesis and respiratory capacity, as well as fatty acid β-oxidation gene expressions in the fat body of the cardiac Sna- overexpressing flies, which is indicative of an increase in lipid utilization that parallels the lean phenotypes exhibited by these flies. Based on these findings, we propose that changes in TG synthesis and breakdown and lipid substrate expenditure in the fat body may underlie the altered systemic lipid levels in the cardiac Sna TF gain- or loss-of-function flies (Fig 8L).

Another observation that we made in the fat body of the cardiac Sna TF-overexpressing or knockdown flies was a decrease or increase, respectively, in the abundance of the Lsd proteins. Lsd1 and Lsd2 are the *Drosophila* homologues of mammalian Perilipin 1 (Plin1) and Perilipin 2 (Plin2), respectively. Both the Lsd and Perilipin proteins are known for their physiological roles in modulating lipid content; for example, the *Plin1*-null mice are lean [52] while the down-modulation of Plin2 alleviates hepatic steatosis [53]. Mirroring the lean phenotypes of their mammalian counterparts, the *lsd1*, *lsd2* double mutant flies’ exhibit reduced TG levels and lipid storage in the fat body [47]. To further understand the causal relationship between fat body Lsd and cardiac Sna TF levels, we inhibited Lsd1 or Lsd2 specifically in the fat body and found an associated development of systemic leanness which phenocopies the cardiac Sna TF-overexpressing flies. Reciprocally, when we overexpressed Lsd1 and/or Lsd2 specifically in the fat body, we detected the development of systemic obese phenotypes that recapitulates those seen in the cardiac Sna TF-inhibited flies. Of note, the fat body-specific overexpression of Lsd2 has also previously been shown to increase whole-body TG levels [44]. Changes in fat body Lsd1 and Lsd2 levels could therefore also contribute to the systemic lipid metabolic phenotypes in the cardiac Sna TF gain- or loss-of-function flies. It will be interesting to investigate in the future the relationship between the Lsd proteins on the lipid droplet surfaces and mitochondrial mass/activity in the fat body of the cardiac Sna TF-overexpressing or inhibited flies, given that Perilipins are known to mediate the physical and metabolic interactions between lipid droplets and mitochondria [54–57].

In summary, our study identifies a previously-unrecognized and unique function of Sna TFs in the heart on normal systemic lipid homeostasis maintenance that involves the regulation of lipolysis and lipogenesis, mitochondrial biogenesis and function, and lipid droplet dynamics in the fat body. Our work in *Drosophila* therefore provides new and striking insights that could advance our understanding of the emerging fields of Sna family protein lipid metabolic functions and heart control of systemic lipid metabolism.

## Materials and methods

### Drosophila culture and strains

All fly stocks were maintained at 25°C on standard medium unless otherwise stated. The following fly lines were used in this study: *UAS-sna*^+^ (FlyORF F000066), *UAS-esg*^+^ (FlyORFF000254), *UAS-wor*^+^ (FlyORF F000155), *UAS-sna*^*RNAi*^ (BDSC BL28679), *UAS-esg*^*RNAi*^ (BDSC BL42846), *UAS-wor*^*RNAi*^ (VDRC v6248), *UAS-lsd1*^*RNAi*^ (BDSC BL65020), *UAS-lsd2*^*RNAi*^ (BDSC BL32846), *UAS-lsd1*^+^ (BDSC BL31784), *UAS-lsd2*^+^ (BDSC BL19733), *Ppl-Gal4* (BDSC BL58768), *R4-Gal4* (BDSC BL 33832), *caudal* (*Cd*)*-Gal4* (BDSC BL3042), *w1118* (BDSC BL 6326), and *tPGH* (BDSC BL 8164). *Tubulin-Gal80*^*ts*^ (BDSC BL7180). *Hand-Gal4* is a gift from Lauren Perrin (AMU, France), and *Myo1A-Gal4* is a gift from Bruce Edgar (University of Utah).

### Temperature shift assays

The overexpression of *UAS* transgenes by *Hand-Gal4* was induced only during the adult phases (from newly-eclosed adults to 1- or 3-week-old adults). To induce transgene overexpression only during the adult stage, parental crosses were kept at 17°C until adult eclosion, after which newly-emerged adult flies were transferred to 29°C for 1 week or 3 weeks before being subjected to systemic metabolic or heart function analyses. These temperature shift experiments were conducted specifically for the overexpression or knockdown of Sna TFs in postnatal hearts only as shown in Fig 4.

### Immunostaining

Tissues were dissected in PBS and either fixed for 20 minutes at room temperature in Bouin's solution (saturated picric acid:formaldehyde:acetic acid in 15:5:1 ratio) (hearts) or in 4% formaldehyde in PBS (fat body and intestine). After three rounds of 10 minute-each washes with 0.1%PBST (0.1%Triton X-100 in PBS), tissues were blocked in 5% goat serum for 1hour at room temperature followed by incubation in primary antibodies in 0.1%PBST at 4°C overnight. The samples were then washed three times, 10 mins each time in 0.1%PBST, followed by 2 hours incubation with alexa-fluor-conjugated secondary antibodies (Jackson Immunoresearch) in 0.1%PBST. After washing as before, tissues were mounted in ProLong Gold antifade reagent with DAPI and viewed under a laser confocal microscope (Olympus FV1000).

The following reagents were used for immunostaining: rabbit anti-Sna at 1:50 (gift from Mark Biggin, Lawrence Berkeley Laboratory), mouse anti-Esg at 1:100 (Abcam ab43635), rat anti- Wor at 1:20 (Abcam 196362), rabbit anti-Lsd1 at 1:100 (gift from Ronald P.Kuhnlein, University of Graz), rabbit anti-Lsd2 at 1:250 (gift from Michael Welte, University of Rochester), mouse anti-ATP5A at 1:500 (gift from Wan-Hee Yoon, OMRF), mouse anti-collagen IV at 1:50 (Abcam ab6586), rabbit anti-Perlecan at 1:1000 (gift from Stefan Baumgartner, Lund University), mouse anti-Pericardin at 1:50 (Developmental Studies Hybridoma Bank EC11), rabbit anti-Nidogen at 1:200 (Anne Holz, University of Giessen), mouse anti-CoVa at 1:50 (Abcam ab13575). Alexa Fluor 488 phalloidin (0.1μM, Thermo Fisher A12379).

### Oil Red O and Bodipy staining

For lipid droplet staining, fat bodies or intestines were dissected in PBS and fixed in 4% paraformaldehyde for 20 min at room temperature. Tissues were then rinsed twice with PBS, incubated for 30 min in either 0.15% Oil Red O (Sigma, O0625), or a 1:500 dilution with PBS of 1 mg/ml Bodipy 493/503 (Invitrogen, D3922). Following incubation, tissues were rinsed three times with PBS. Stained samples were mounted in ProLongTM Gold antifade reagent with DAPI and imaged by Olympus Confocal Microscope. Quantification of the size (perimeter) of Oil Red O-stained lipid droplets were performed using the ImageJ software, with 40 lipid droplets analyzed from six fat bodies per genotype. Data was analyzed using Graphpad Prism 8.

### Western Blotting

Fat bodies were collected and homogenized with 0.1% Triton-X100 and phosphatase and protease inhibitors (Sigma, P2714). Samples of ~30 μg protein were resolved in SDS-PAGE gels (Invitrogen) and transferred to nitrocellulose membranes. Membranes were incubated overnight at 4°C with the following primary antibodies at the indicated dilutions: rabbit anti-Akt (pan) at 1:1000 (Cell Signaling), rabbit anti-pAKT (Ser505) at 1:1000 (Cell Signaling), rabbit anti-Lsd1 at 1:1500 and rabbit anti-Lsd2 at 1:1500 (see their descriptions above), mouse anti- tubulin at 1:5000 (Sigma). After washing the membranes for 3 times at 20 mins each, membranes were incubated with horseradish peroxidase-conjugated anti-mouse and anti-rabbit secondary antibodies (Perkin Elmer) and bands were visualized with ECLTM Western Blotting Detection Reagents (GE Healthcare, RPN2209). The intensity of band was quantified by Image J from three independent results.

### Insulin treatment

For insulin sensitivity experiment, fat bodies were incubated with human insulin (1 μM final concentration) for 15 mins followed by homogenization with 0.05%PBST with protease and phosphatase inhibitor. Samples were centrifuged and the supernatants subjected to Western blot analyses as described above.

### TUNEL labeling

TUNEL labeling kit (Roche, Basel, Switzerland) was used to label apoptotic cells. Adult heart tissues were fixed with freshly prepared 4% PFA fixation solution for 20 mins at RT. Samples were rinsed with PBS for 3 times, and Incubated with Blocking solution (5% donkey serum) for 10 mins at RT. After rinsing with PBS, samples were incubated in permeabilization solution (0.1% PBST, PBS+0.1% Triton) for 2mins on ice. Rinsing twice with PBS, 50μl of TUNEL labeling solution was added to samples and incubated at 37°C for 1hr. Samples were rinsed 3 times with PBS and mounted in ProLong Gold antifade reagent with DAPI.

### Quantitative RT-PCR

For fat body and heart samples, total RNA was extracted with Trizol reagent (ThermoFisher, 15596026). cDNA was synthesized from total RNA with oligo(dT) and superscript III reverse transcriptase (ThermoFisher, 18080085). DNA was amplified using PCR master mix (ThermoFisher, 4364346) and the following PCR conditions was used: 3min at 95 °C, (30 sec at 95°C, 20 sec at 57°C, and 30 sec at 72°C) × 39, and 10 min at 72°C. Values were normalized with *RpL14*. Primers used are shown in S1 Table.

### Triglyceride assay

Triglyceride assay was performed as previously described [9]. Briefly, adult flies Flies (12 per genotype, 1:1 ratio of males:females) were weighed and homogenized in PBS containing 0.1% Triton-X100 in an amount (μl) that is 8 X the total weight of flies (μg). The homogenates were centrifuged and the supernatants were removed and incubated with Infinity Triglyceride Reagent (Thermo Electron) for 30 min at 37°C. The absorbance at 540 nm was then measured and TG content was calculated from a standard curve constructed with solutions of known TG concentrations (Thermo Electron). The results were normalized to the protein concentration (μg/μl) of each sample with the Bradford assay (5000002, Bio-Rad).

### Food intake assay

Food intake for adult flies was measured using the CApillary FEeder assay (CAFE) [58] Briefly, 1-week old adult female flies were starved for 16 hours before being introduced into fresh vials containing liquid food: 160μL blue dye solution + 320μL 1M sucrose + 320 μL distilled water in 100 μL (1 μL /mm) capillary tubes (10 flies per vial). Liquid food uptake was measured at the end of 6 hour feeding. A control liquid food vial containing no flies was used for the correction of liquid food evaporation. Food intake (μl food) was normalized to weight (g).

### Tetramethylrhodamine ethyl ester (TMRE) staining

Mitochondria membrane potential was detected using the Tetramethylrhodamine ethyl ester (TMRE) staining as previously described [59]. Briefly, adult fly abdomens were dissected and incubated with freshly-prepared solution of 20 nM TMRE (ThermoFisher T669) in in the dark for 5 to 7 min. Abdomens were then washed briefly twice in PBS and immediately analyzed under an Olympus FV1000 confocal microscope with a 100x oil objective.

### Semi-intact drosophila heart preparation and heartbeat analysis

Cardiac contractility measurements on semi-intact preparations of adult fly hearts were performed as described previously [60]. High-speed 30-s movies were recorded at a rate of *>*150 frames per second using a Hamamatsu CCD camera on a Nikon 80i upright microscope with a 10x dipping immersion lens. The images were processed using Simple PCI imaging software (Compix Inc.). Quantitative data were generated using a MatLab-based image analysis program.

### ATP production

Intracellular ATP levels were measured by the Celltiter-Glo kit (Promega, G7553) following the manufacturer’s instructions. Briefly, fat bodies from 10 adult flies were homogenized in 50 μL of extraction buffer (6 M Guanidine Chloride, 100 mM Tris·HCl pH 8.0, 4 mM EDTA), and immediately heated at 70 °C for 5 min. Samples were then centrifuged at 12000rpm for 10 min at 4 °C. 10μl of the supernatant was added to each well of a fresh 96-well plate with 40μl water pre-added. 50μl of the reaction mix (provided in kit) was then added to each well. The level of ATP production was measured using the BioTek Multimode reader with Gene5 software and 114 Lum/1536 Lum filter. ATP concentration was further normalized to protein level that was measured by the Bradford Reagent (Bio-Rad, 5000006).

### Oxygen consumption rate

Fat body oxygen consumption rate (OCR) was assayed by the Seahorse XFe96 (Agilent) at 25°C as described in [61]. Briefly, adult fat bodies were dissected in PBS and transferred to 96 well Islet Capture Microplate with 175μL assay medium (*Drosophila* S2 cell medium with 2 mM sodium pyruvate). Oligomycin was injected at a final concentration of 10μM, followed by FCCP at a final concentration of 0.4μM, and finally Rotenone/Antimycin (RAA) at a final concentration of 3 μM Rotenone and 12μM Antimycin. Data was analyzed using Prism Graphpad7 and represent the average OCR of n>10 wells of fat body for each genotype. Basal respiration was calculated as OCR before oligomycin minus OCR after antimycin. Maximal respiration was calculated as OCR after FCCP minus OCR after antimycin.

### Transmission electron microscopy

Drosophila fat samples were fixed with 4% Paraformaldehyde (EM grade), 2% Gluteraldehyde (EM grade), in 0.1M Sodium Cacodylate buffer overnight on a rocker at room temperature. Samples were then post fixed for 90 minutes in 1% Osmium tetroxide (OsO4) in 0.1M Sodium Cacodylate, and rinsed three times for five minutes each in 0.1M Sodium Cacodylate buffer. The samples were then dehydrated in a graded acetone series. The acetone gradient was as follows: 50%, 60%, 75%, 85%, 95%, 100%. The samples were in each concentration for 15 minutes on a rocker at room temperature. Then the samples had two 15 minute treatments in 100% Propylene Oxide. Following dehydration, the samples were infiltrated in a graded Epon/Araldite (EMS) resin /Propylene Oxide series (1:4. 1:3, 1:1, 3:1) for 60 minutes, 60 minutes, overnight, and 120 minutes the next day respectfully. The samples were further infiltrated with pure resin for 45 minutes, 90 minutes, and then overnight. The samples were then embedded in resin plus BDMA (accelerator) and polymerized at 60°C for 48 hours. Ultrathin sections were stained with Sato’s Lead and Saturated Uranyl Acetate in 50% methanol before viewing on a Hitachi H7600 Transmission Electron Microscope.

### Point counting grids

To quantify mitochondrial volume density, a square lattice grid of 100 points was digitally projected over each electron micrograph. This was followed by counting the number of times a mitochondria is located at a point. The number of times a mitochondria is located at a point is then divided by the total number of points (>3000) and expressed as a percent yield that represents the mitochondrial volume density (% of cell volume occupied by mitochondria).

### Statistical analysis

All data are presented as the mean ± SEM of the indicated number of replicates. Data were analyzed using the two-tailed Student’s *t*-test and *p* < 0.05 was considered statistically significant. Student’s two-tailed unpaired *t*-test assuming equal variance.

## Supporting information

S1 Fig *Hand-Gal4* drives expression specifically in cardiomyocytes and not fat body or intestine. (A-A’) Anatomical schematic (A) of an adult *Drosophila* heart comprising a linear tube of two rows of cardiomyocytes (green) with an inner lumen through which the hemolymph pumps. The cardiomyocyte tube is surrounded by two outer rows of non-muscle pericardial cells (grey), which serve as supporting cells to the cardiomyocytes. (A’) Representative confocal image of an adult *Hand>GFP* heart tube at low magnification (20X) showing GFP (green) expression throughout the heart tube. Scale bar represents 50 μm. (B-D) Representative confocal images of GFP (green) immunostainings and DAPI (blue) co- stainings in the heart (B), fat body (C), or intestine (D) in flies bearing *Hand-Gal4* and *UAS-GFP* constructs (*Hand>GFP*). Scale bar represents 20 μm. Dotted lines mark the outlines of heart tube (B), fat body (C), or intestinal tube (D).
(TIF)

S2 Fig Cardiac-specific inhibition of Snail TFs perturbs systemic lipid parameters. (A-C) Relative mRNA level of Sna TF genes *sna* (A), *esg* (B), and *wor* (C) in adult hearts of control flies (*Hand-Gal4*) and flies with cardiomyocyte-specific knockdown of Sna TF genes (*Hand>esg*^*RNAi*^; *sna*^*RNAi*^ and *Hand>esg*^*RNAi*^; *wor*^*RNAi*^). Results are the mean ± SEM of 60 hearts analyzed for 3 independent experiments and are expressed as the fold change compared with control hearts (set to 1.0). (D-D’) Abdominal fat mass in 7-day old control flies (*Hand-Gal4*) (D) and flies with cardiomyocyte-specific inhibition of Sna TFs (*Hand>esg*^*RNAi*^; *sna*^*RNAi*^) (D’). (E-F) Fat body (E) and intestine (F) TG level of 7-day old control flies (*Hand-Gal4*), and flies with cardiomyocyte-specific knockdown of Snail TF genes (*Hand>esg*^*RNAi*^; *sna*^*RNAi*^ and *Hand>esg*^*RNAi*^; *wor*^*RNAi*^). TG levels (μg/μl) were normalized to total protein (μg/μl). Results are the mean ± SEM of 30–40 flies analyzed over at least 3 independent experiments and are expressed as the fold change normalized TG compared with that of the control flies (set to 1.0). (G) Western blot analyses of phosphorylated Akt (pAKT) and total Akt (AKT) in the fat bodies of 1-week old control flies (*Hand-Gal4*) and flies with cardiomyocyte-specific inhibition of Sna TFs (*Hand>esg*^*RNAi*^; *sna*^*RNAi*^) in the absence or presence of insulin stimulation. α-Tubulin was used as loading control. About thirty μg of protein was loaded per lane. (G’) Quantification of the ratio pf phosphorylated Akt (pAkt) to total Akt (tAkt) in the fat bodies of1-week old control flies (*Hand-Gal4*) and flies with cardiomyocyte-specific inhibition of Sna TFs (*Hand>esgRNAi*; *snaRNAi*) in the presence of insulin stimulation. Results are the mean ± SEM of 20 fat bodies analyzed for 3 independent experiments and are expressed as the fold change compared with control fat bodies (set to 1.0).
(TIF)

S3 Fig *Hand-Gal4*–mediated overexpression or knockdown of Snail TFs alters Snail TF levels in the heart but not fat body. (A-C’) Representative confocal images of 7-day-old hearts immunostained for Sna (A-A’), Esg (B-B’), or Wor (C-C’) and co-stained with DAPI (blue) in control flies (*Hand-Gal4*) (A, B, C) and in flies with cardiomyocyte-specific overexpression of Sna (*Hand>sna*^+^) (A’), Esg (*Hand>esg*^+^) (B’), or Wor (*Hand>wor*^+^) (C’). For each genotype, 6 hearts were analyzed. Dotted lines mark the outlines of heart tubes. Scale bar represents 20 μm. Arrows in A’, B’, and C’ indicate the respective localizations of Sna (A’), Esg (B’), and Wor (C’) to nuclei in the cardiomyocytes. (D-F’) Representative confocal images of 7-day-old fat bodies immunostained for Sna (D-D’), Esg (E-E’), or Wor (F-F’), and co-stained with DAPI (blue) in control flies (*Hand-Gal4*) (D, E, F) and flies with cardiomyocyte-specific overexpression of Sna (*Hand>sna*^+^) (D’, E’, F’).
(TIF)

S4 Fig Cardiac-specific overexpression of Snail TFs perturbs systemic lipid parameters. (A-A”‘) Abdominal fat mass in 7-day old control flies (*Hand-Gal4*) (A) and flies with cardiomyocyte-specific overexpression of Sna (*Hand>sna*^+^) (A’), Wor (*Hand>wor*^+^) (A”), or Esg (*Hand>esg*^+^) (A”‘). White arrows denote gaps in fat mass. (B) Western blot analyses of phosphorylated Akt (pAKT) and total Akt (AKT) in the fat bodies of 1-week old control flies (*Hand-Gal4*) and flies with cardiomyocyte-specific overexpression of Sna (*Hand>sna*^+^) in the absence or presence of insulin stimulation. α-Tubulin was used as a loading control. About thirty μg of protein was loaded per lane. (B’) Quantification of the ratio pf phosphorylated Akt (pAkt) to total Akt (tAkt) in the fat bodies of1-week old control flies (*Hand-Gal4*) and flies with cardiomyocyte-specific overexpression of Sna (*Hand>sna*^+^) in the presence of insulin stimulation. Results are the mean ± SEM of 20 fat bodies analyzed for 3 independent experiments and are expressed as the fold change compared with control fat bodies (set to 1.0). (C-D’) Representative confocal images of GFP immunostaining (green) and DAPI co-staining (blue) in the fat bodies of1-week old *tGPH* flies bearing *Hand-Gal4* (*Hand-Gal4;tGPH*) (C-C’) or *tGPH* flies with cardiomyocyte-specific overexpression of Sna (*Hand>sna*^+^;*tGPH*) (D-D’) under insulin stimulation. Scale bar represents 20 μm. (E-E”‘) Representative confocal images of Sna immunostaining (red) and DAPI co-staining (blue) in the fat bodies of 1-week old control flies (*Hand-Gal4*) (E-E’) and flies with cardiomyocyte-specific overexpression of Sna (*Hand>sna*^+^) (E”) or knockdown of Sna TFs (*Hand>esg*^*RNAi*^; *sna*^*RNAi*^) (E”‘) in the presence (E’-E”‘) or absence (E) of insulin stimulation. Scale bar represents 20 μm. (F) Quantification of Sna immunofluorescence in fat bodies from control flies (*Hand-Gal4*), flies bearing the cardiac-specific overexpression of Sna (*Hand>sna*^+^), and flies with cardiomyocyte- specific inhibition of Sna TFs (*Hand>esg*^*RNAi*^; *sna*^*RNAi*^) without or with insulin stimulation. Three separate areas were analyzed from each of 5 fat bodies per genotype and results are represented as mean ± SEM and expressed as the fold change compared with control fat bodies (set to 1.0).
(TIF)

S5 Fig Fat body- or intestinal manipulations of Snail TFs does not alter systemic lipid parameters. (A-B) Whole-body TG level of 7-day old control flies [*Ppl-Gal4* (A) or *R4-Gal4* (B)], flies with fat body-specific overexpression of Sna [*Ppl>sna*^+^ (A) or *R4>sna*^+^ (B)], and flies with fat body- specific knockdown of Sna TFs (*Ppl>esg*^*RNAi*^; *sna*^*RNAi*^ (A) or *R4*> *esg*^*RNAi*^; *sna*^*RNAi*^ (B)]. TG levels (μg/μl) were normalized to total protein (μg/μl). Results are the mean ± SEM of 30–40 flies analyzed over at least 3 independent experiments and are expressed as the fold change normalized TG compared with that of the control flies (set to 1.0). (C-D) Whole-body TG level of 7-day old control flies [*Cd-Gal4* (C) or *Myo1A-Gal4* (D)], flies with intestinal-specific overexpression of Sna [*Cd>sna*^+^ (C) or *Myo1A>sna*^+^ (D)], and flies with intestinal-specific knockdown of Sna TFs (*Cd>esg*^*RNAi*^; *sna*^*RNAi*^ (C) or *Myo1A*> *esg*^*RNAi*^; *sna*^*RNAi*^ (D)]. TG levels (μg/μl) were normalized to total protein (μg/μl). Results are the mean ± SEM of 30–40 flies analyzed over at least 3 independent experiments and are expressed as the fold change normalized TG compared with that of the control flies (set to 1.0). (E-E”) Representative confocal images of hearts stained with Bodipy (green) and DAPI (blue) from 7-day old control flies (*Ppl-Gal4*) (E), flies with fat body-specific overexpression of Sna (*Hand>sna*^+^) (E’), and flies with fat body-specific knockdown of Sna TF genes (*Hand>esg*^*RNAi*^; *sna*^*RNAi*^) (E”). For each genotype, 6 hearts were analyzed. Dotted lines mark the outlines of heart tubes. Scale bar represents 20 μm. (F-F”) Representative confocal images of fat bodies stained with Oil Red O (red) and DAPI (blue) from 7-day old control flies (*Ppl-Gal4*) (F), flies with fat body-specific overexpression of Sna (*Ppl>sna*^+^) (F’), and flies with fat body-specific knockdown of Sna TF genes (*Ppl>esg*^*RNAi*^; *sna*^*RNAi*^) (F”). For each genotype, 6 fat bodies were analyzed. Dotted lines mark the outlines of fat bodies. Scale bar represents 20 μm.
(TIF)

S6 Fig Overexpression or knockdown of Sna TF genes in postnatal hearts only perturbs intestinal triglyceride content and abdominal fat mass. (A-A’) Representative confocal images of hearts immunostained for Sna (red) and co- stained with DAPI (blue) from newly-eclosed control flies (*Tub-Gal80*^*ts*^; *Hand-Gal4*) or flies bearing *Tub-Gal80*^*ts*^, *Hand-Gal4*, and *UAS-sna*^+^ (*Tub-Gal80*^*ts*^; *Hand>sna*^+^) that have been reared from embryo to newly-eclosion at 17°C. For each genotype, 6 hearts were analyzed. Dotted lines mark the outlines of heart tubes. Scale bar represents 20 μm. (B-B’) Representative confocal images of hearts immunostained for Sna (red) and co- stained with DAPI (blue) from 7-day-old control flies (*Tub-Gal80*^*ts*^; *Hand-Gal4*) and flies bearing *Tub-Gal80*^*ts*^, *Hand-Gal4*, and *UAS-sna*^+^ (*Tub-Gal80*^*ts*^; *Hand>sna*^+^) that have been reared from embryo to newly-eclosion at 17°C and from newly-eclosion to 7-day-old adulthood at 29°C. For each genotype, 6 hearts were analyzed. Dotted lines mark the outlines of heart tubes. Arrow denotes Sna localizations to nuclei in the cardiomyocytes. Scale bar represents 20 μm. (C) Intestinal TG level of 7-day old control flies (*Tub-Gal80*^*ts*^; *Hand-Gal4*) and flies with cardiomyocyte-specific overexpression of Snail TFs in postnatal hearts only (*Tub-Gal80*^*ts*^; *Hand>sna*^+^ or *Tub-Gal80*^*ts*^; *Hand>wor*^+^ or *Tub-Gal80*^*ts*^; *Hand>esg*^+^). TG levels (μg/μl) were normalized to total protein (μg/μl). Results are the mean ± SEM of 30–40 flies analyzed over at least 3 independent experiments and are expressed as the fold change normalized TG compared with that of the control flies (set to 1.0). (D-D”‘) Abdominal fat mass in 7-day old control flies (*Tub-Gal80*^*ts*^; *Hand-Gal4*) (D) and flies that overexpress Sna TFs in postnatal hearts only (*Tub-Gal80*^*ts*^; *Hand>sna*^+^ or *Tub-Gal80*^*ts*^; *Hand>wor*^+^ or *Tub-Gal80*^*ts*^; *Hand>esg*^+^) (D’-D”‘). White arrows denote gaps in fat mass. (E-E’) Abdominal fat mass in 7-day old control flies (*Tub-Gal80*^*ts*^; *Hand-Gal4*) (E) and flies with Sna TF gene knockdowns in postnatal hearts only (*Tub-Gal80*^*ts*^; *Hand>esg*^*RNAi*^, sna^*RNAi*^) (E’).
(TIF)

S7 Fig Cardiac-specific manipulations of Snail TFs do not affect cardiac lipid metabolism and fly food intake. (A-F) Relative mRNA level of FFA synthesis gene *Fas* (A), TG lipogenesis genes *AGPAT3* (B) and *Lipin* (C), lipolytic gene *Bmm* (D), mitochondrial biogenesis gene *PGC-1*/*spargel* (E), and lipid droplet surface protein gene *Lsd2* (F) in hearts of control flies (*Hand-Gal4*) and flies with cardiomyocyte-specific knockdown of Sna TF genes (*Hand>esg*^*RNAi*^; *sna*^*RNAi*^). Results are the mean ± SEM of 30 hearts analyzed over 3 independent experiments and are expressed as the fold change compared with control hearts (set to 1.0). (G-H) CAFÉ assay of food intake in 1-wk old control flies (*Hand-Gal4*) and flies with cardiomyocyte-specific overexpression of Sna (*Hand>sna*^+^), Wor (*Hand>wor*^+^), or Esg (*Hand>esg*^+^) (G), or in 1-wk old control flies (*Hand-Gal4*) and flies with cardiomyocyte-specific inhibition of Sna TFs (*Hand>esg*^*RNAi*^; *wor*^*RNAi*^ or *Hand>esg*^*RNAi*^; *sna*^*RNAi*^) (H). Results are the mean ± SEM of 30 flies analyzed over at least 3 independent experiments. (I) Steady-state ATP level in the fat bodies of 7-day-old control flies (*Hand-Gal4*) and flies with cardiomyocyte-specific knockdown of Snail TF genes (*Hand>esg*^*RNAi*^; *sna*^*RNAi*^). Results are the mean ± SEM of fat bodies isolated from 30–40 flies analyzed over 3 independent experiments and are expressed as the fold change compared with control hearts (set to 1.0).
(TIF)

S8 Fig Cardiac-specific overexpression of Sna up-regulates mitochondrial β-oxidation gene expression in fat body. (A-C) Relative mRNA level of genes encoding acyl-coA dehydrogenases (CG17544 and CG9527) (A-B) and gene encoding acyl-coA oxidase 57D-p (CG9707) (C) in fat bodies of 7-day- old control flies (*Hand-Gal4*) and flies with cardiomyocyte-specific overexpression of Sna (*Hand>sna*^+^). Results are the mean ± SEM of 30–40 fat bodies analyzed over 3 independent experiments and are expressed as the fold change compared with control fat bodies (set to 1.0).
(TIF)

S9 Fig Alterations in fat body Lsd1 and Lsd2 levels evoke systemic lipid metabolic effects. (A-B) Whole-body TG level (A) and fat body TG level (B) of 7-day old control flies (Ppl*-Gal4*) and flies with fat body-specific inhibition of Lsd1 (*Ppl>Lsd1*^*RNAi*^) or Lsd2 (*Ppl>Lsd2*^*RNAi*^). TG levels (μg/μl) were normalized to total protein (μg/μl). Results are the mean ± SEM of 30–40 flies analyzed over at least 3 independent experiments and are expressed as the fold change normalized TG compared with that of the control flies (set to 1.0). (C-C’) Representative confocal images of abdominal fat bodies stained with Oil Red O (red) and DAPI (blue) from 1-wk-old control flies and flies with Lsd2 knockdown in fat body. For each genotype, at least 6 fat bodies were analyzed. Scale bar represents 20 μm. (D-E) Whole-body TG level (A) and fat body TG level (B) of 7-day old control flies (Ppl*-Gal4*) and flies with fat body-specific overexpression of Lsd1 (*Ppl>Lsd*^+^) or Lsd2 (*Ppl>Lsd2*^+^) or both (*Ppl>Lsd*^+^;*Lsd2*^+^). TG levels (μg/μl) were normalized to total protein (μg/μl). Results are the mean ± SEM of 30–40 flies analyzed over at least 3 independent experiments and are expressed as the fold change normalized TG compared with that of the control flies (set to 1.0). (F-F’) Representative confocal images of abdominal fat bodies stained with Oil Red O (red) and DAPI (blue) from 1-wk-old control flies (F) and flies with Lsd1 and Lsd2 co-overexpression in fat body (*Ppl>Lsd*^+^;*Lsd2*^+^) (F’). For each genotype, at least 6 fat bodies were analyzed. Scale bar represents 20 μm.
(TIF)

S1 Table List of primers used in all qRT-PCR experiments in this study. (TIF)
