# Supplementary figures and images for: Cardiac Snail family of transcription factors directs systemic lipid metabolism in Drosophila
Source: PLoS Genet. 2019 Nov 14;15(11):e1008487. doi: 10.1371/journal.pgen.1008487 (PMC6879157; doi:10.1371/journal.pgen.1008487)

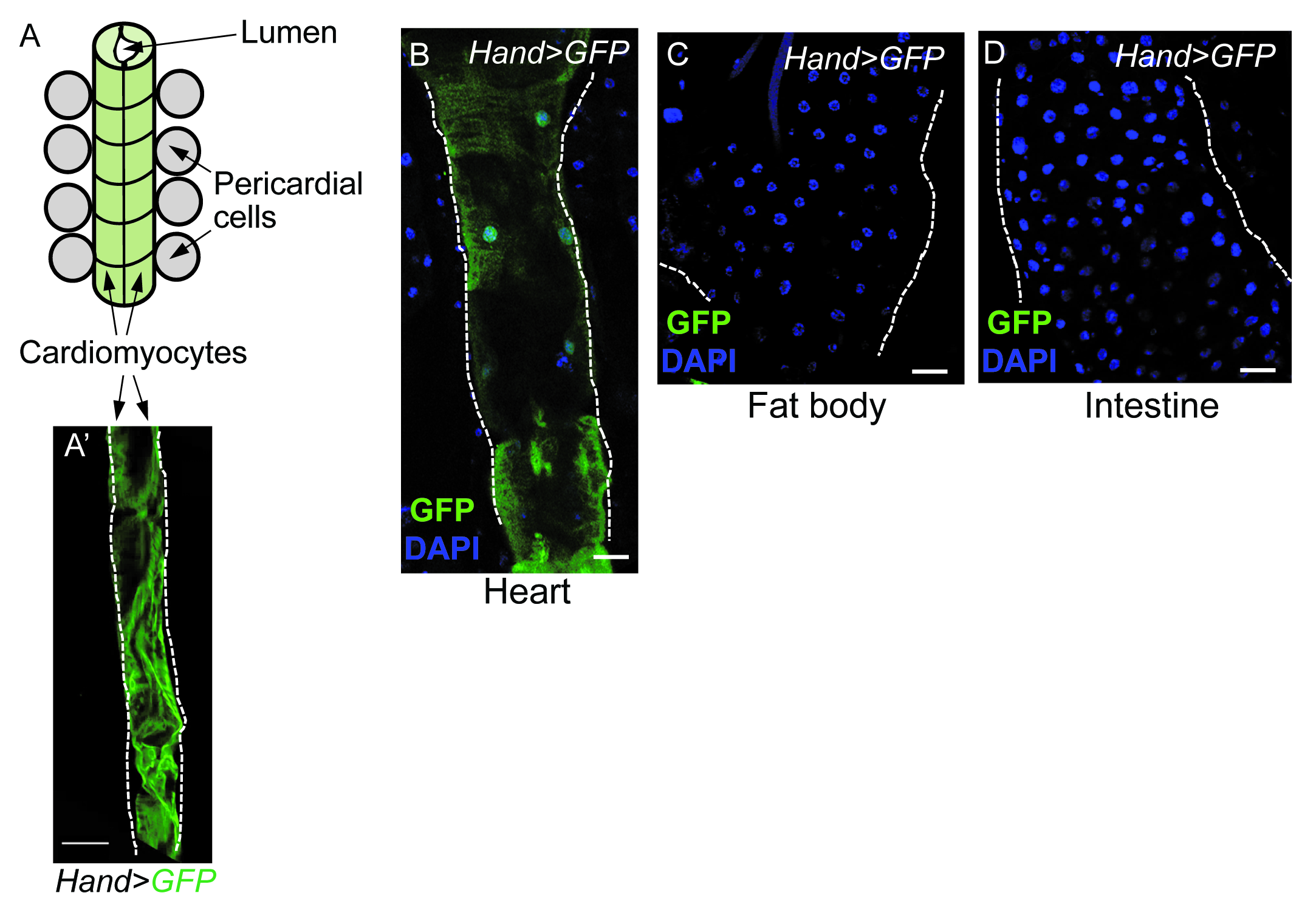

Supplement: S1 Fig — (A-A’) Anatomical schematic (A) of an adult Drosophila heart comprising a linear tube of two rows of cardiomyocytes (green) with an inner lumen through which the hemolymph pumps. The cardiomyocyte tube is surrounded by two outer rows of non-muscle pericardial cells (grey), which serve as supporting cells to the cardiomyocytes. (A’) Representative confocal image of an adult Hand>GFP heart tube at low magnification (20X) showing GFP (green) expression throughout the heart tube. Scale bar represents 50 μm. (B-D) Representative confocal images of GFP (green) immunostainings and DAPI (blue) co- stainings in the heart (B), fat body (C), or intestine (D) in flies bearing Hand-Gal4 and UAS-GFP constructs (Hand>GFP). Scale bar represents 20 μm. Dotted lines mark the outlines of heart tube (B), fat body (C), or intestinal tube (D). (TIF) [file pgen.1008487.s001.tif]

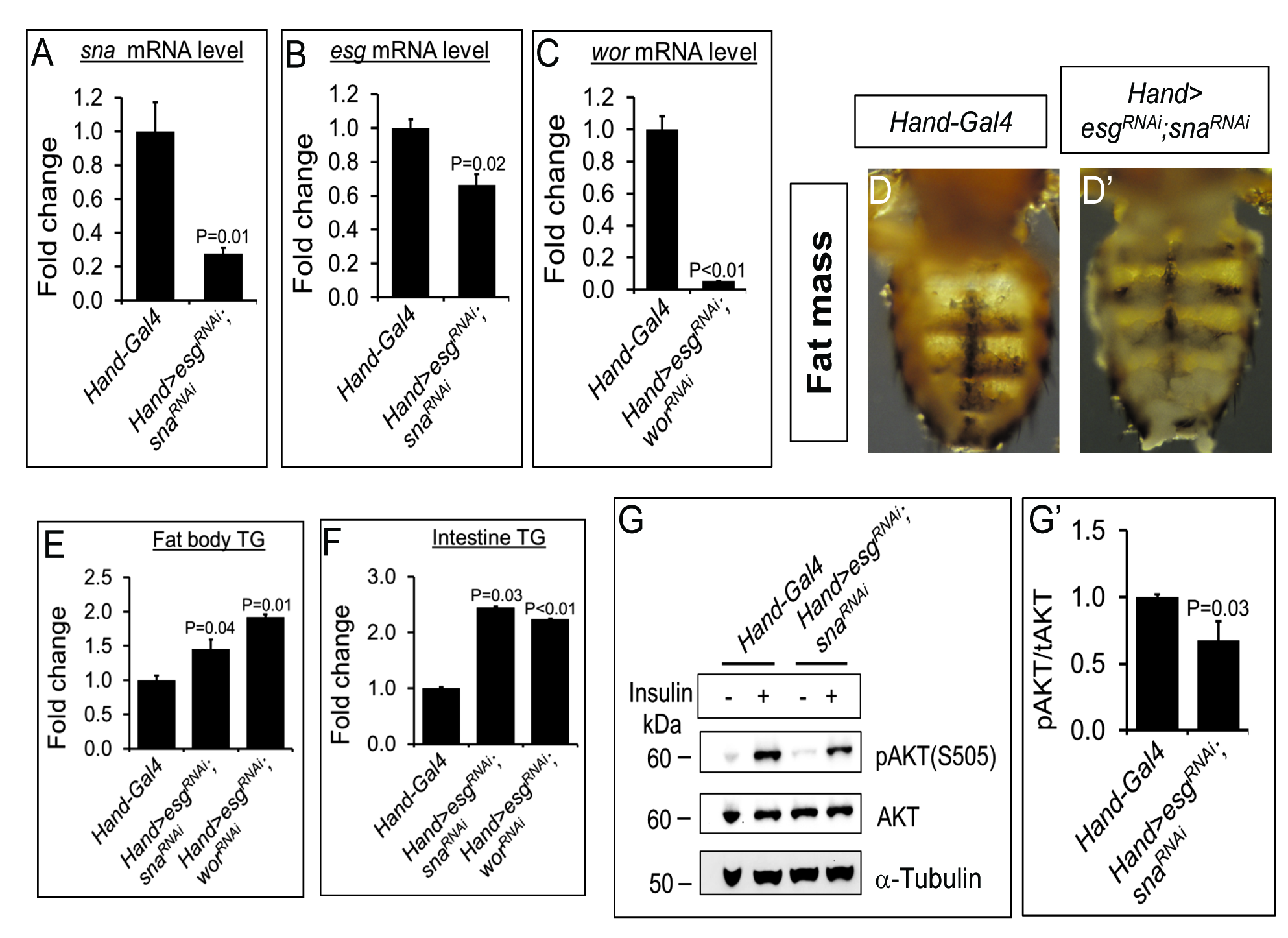

Supplement: S2 Fig — (A-C) Relative mRNA level of Sna TF genes sna (A), esg (B), and wor (C) in adult hearts of control flies (Hand-Gal4) and flies with cardiomyocyte-specific knockdown of Sna TF genes (Hand>esgRNAi; snaRNAi and Hand>esgRNAi; worRNAi). Results are the mean ± SEM of 60 hearts analyzed for 3 independent experiments and are expressed as the fold change compared with control hearts (set to 1.0). (D-D’) Abdominal fat mass in 7-day old control flies (Hand-Gal4) (D) and flies with cardiomyocyte-specific inhibition of Sna TFs (Hand>esgRNAi; snaRNAi) (D’). (E-F) Fat body (E) and intestine (F) TG level of 7-day old control flies (Hand-Gal4), and flies with cardiomyocyte-specific knockdown of Snail TF genes (Hand>esgRNAi; snaRNAi and Hand>esgRNAi; worRNAi). TG levels (μg/μl) were normalized to total protein (μg/μl). Results are the mean ± SEM of 30–40 flies analyzed over at least 3 independent experiments and are expressed as the fold change normalized TG compared with that of the control flies (set to 1.0). (G) Western blot analyses of phosphorylated Akt (pAKT) and total Akt (AKT) in the fat bodies of 1-week old control flies (Hand-Gal4) and flies with cardiomyocyte-specific inhibition of Sna TFs (Hand>esgRNAi; snaRNAi) in the absence or presence of insulin stimulation. α-Tubulin was used as loading control. About thirty μg of protein was loaded per lane. (G’) Quantification of the ratio pf phosphorylated Akt (pAkt) to total Akt (tAkt) in the fat bodies of1-week old control flies (Hand-Gal4) and flies with cardiomyocyte-specific inhibition of Sna TFs (Hand>esgRNAi; snaRNAi) in the presence of insulin stimulation. Results are the mean ± SEM of 20 fat bodies analyzed for 3 independent experiments and are expressed as the fold change compared with control fat bodies (set to 1.0). (TIF) [file pgen.1008487.s002.tif]

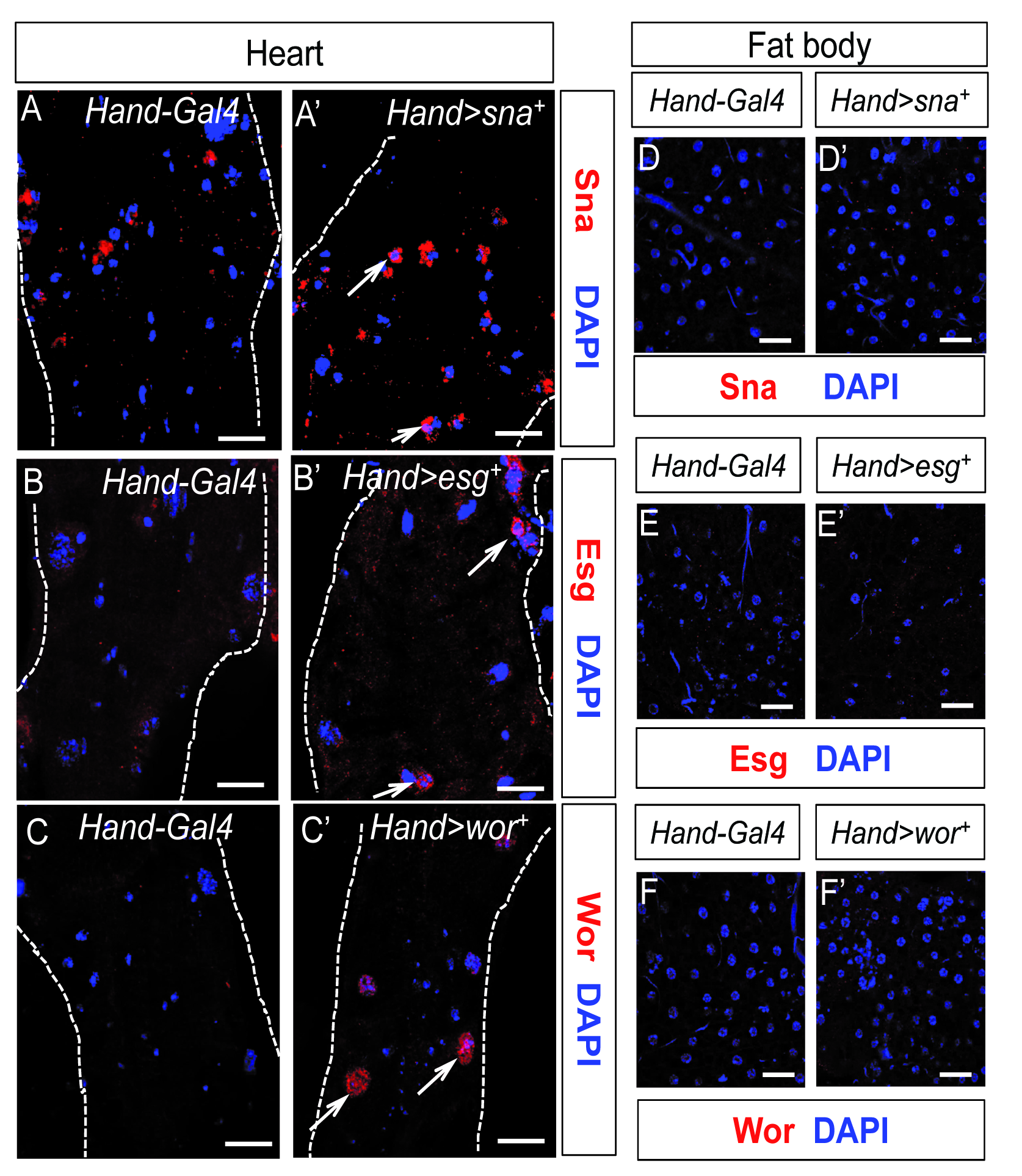

Supplement: S3 Fig — (A-C’) Representative confocal images of 7-day-old hearts immunostained for Sna (A-A’), Esg (B-B’), or Wor (C-C’) and co-stained with DAPI (blue) in control flies (Hand-Gal4) (A, B, C) and in flies with cardiomyocyte-specific overexpression of Sna (Hand>sna+) (A’), Esg (Hand>esg+) (B’), or Wor (Hand>wor+) (C’). For each genotype, 6 hearts were analyzed. Dotted lines mark the outlines of heart tubes. Scale bar represents 20 μm. Arrows in A’, B’, and C’ indicate the respective localizations of Sna (A’), Esg (B’), and Wor (C’) to nuclei in the cardiomyocytes. (D-F’) Representative confocal images of 7-day-old fat bodies immunostained for Sna (D-D’), Esg (E-E’), or Wor (F-F’), and co-stained with DAPI (blue) in control flies (Hand-Gal4) (D, E, F) and flies with cardiomyocyte-specific overexpression of Sna (Hand>sna+) (D’, E’, F’). (TIF) [file pgen.1008487.s003.tif]

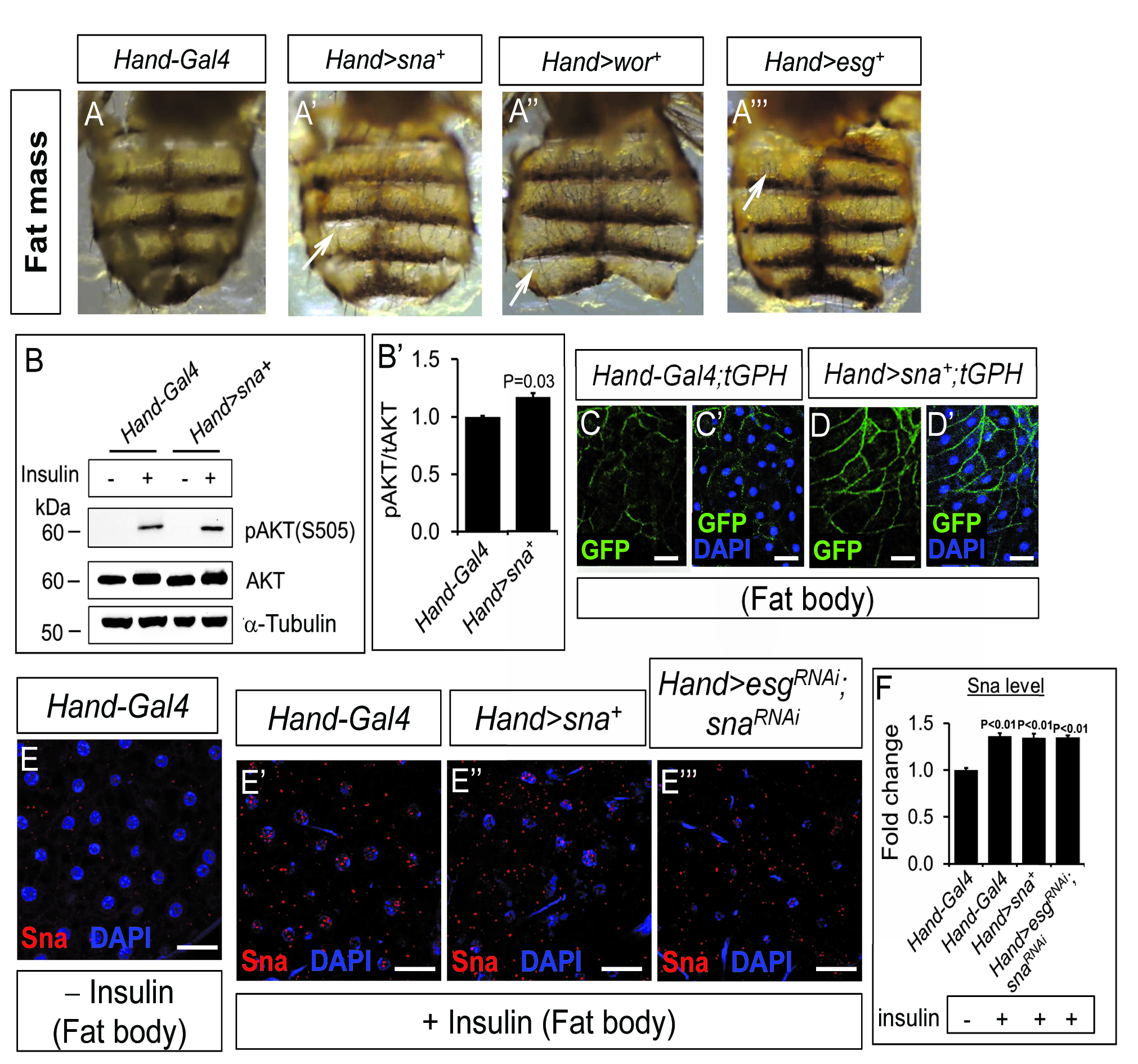

Supplement: S4 Fig — (A-A”‘) Abdominal fat mass in 7-day old control flies (Hand-Gal4) (A) and flies with cardiomyocyte-specific overexpression of Sna (Hand>sna+) (A’), Wor (Hand>wor+) (A”), or Esg (Hand>esg+) (A”‘). White arrows denote gaps in fat mass. (B) Western blot analyses of phosphorylated Akt (pAKT) and total Akt (AKT) in the fat bodies of 1-week old control flies (Hand-Gal4) and flies with cardiomyocyte-specific overexpression of Sna (Hand>sna+) in the absence or presence of insulin stimulation. α-Tubulin was used as a loading control. About thirty μg of protein was loaded per lane. (B’) Quantification of the ratio pf phosphorylated Akt (pAkt) to total Akt (tAkt) in the fat bodies of1-week old control flies (Hand-Gal4) and flies with cardiomyocyte-specific overexpression of Sna (Hand>sna+) in the presence of insulin stimulation. Results are the mean ± SEM of 20 fat bodies analyzed for 3 independent experiments and are expressed as the fold change compared with control fat bodies (set to 1.0). (C-D’) Representative confocal images of GFP immunostaining (green) and DAPI co-staining (blue) in the fat bodies of1-week old tGPH flies bearing Hand-Gal4 (Hand-Gal4;tGPH) (C-C’) or tGPH flies with cardiomyocyte-specific overexpression of Sna (Hand>sna+;tGPH) (D-D’) under insulin stimulation. Scale bar represents 20 μm. (E-E”‘) Representative confocal images of Sna immunostaining (red) and DAPI co-staining (blue) in the fat bodies of 1-week old control flies (Hand-Gal4) (E-E’) and flies with cardiomyocyte-specific overexpression of Sna (Hand>sna+) (E”) or knockdown of Sna TFs (Hand>esgRNAi; snaRNAi) (E”‘) in the presence (E’-E”‘) or absence (E) of insulin stimulation. Scale bar represents 20 μm. (F) Quantification of Sna immunofluorescence in fat bodies from control flies (Hand-Gal4), flies bearing the cardiac-specific overexpression of Sna (Hand>sna+), and flies with cardiomyocyte- specific inhibition of Sna TFs (Hand>esgRNAi; snaRNAi) without or with insulin stimulation. Three separate [file pgen.1008487.s004.tif]

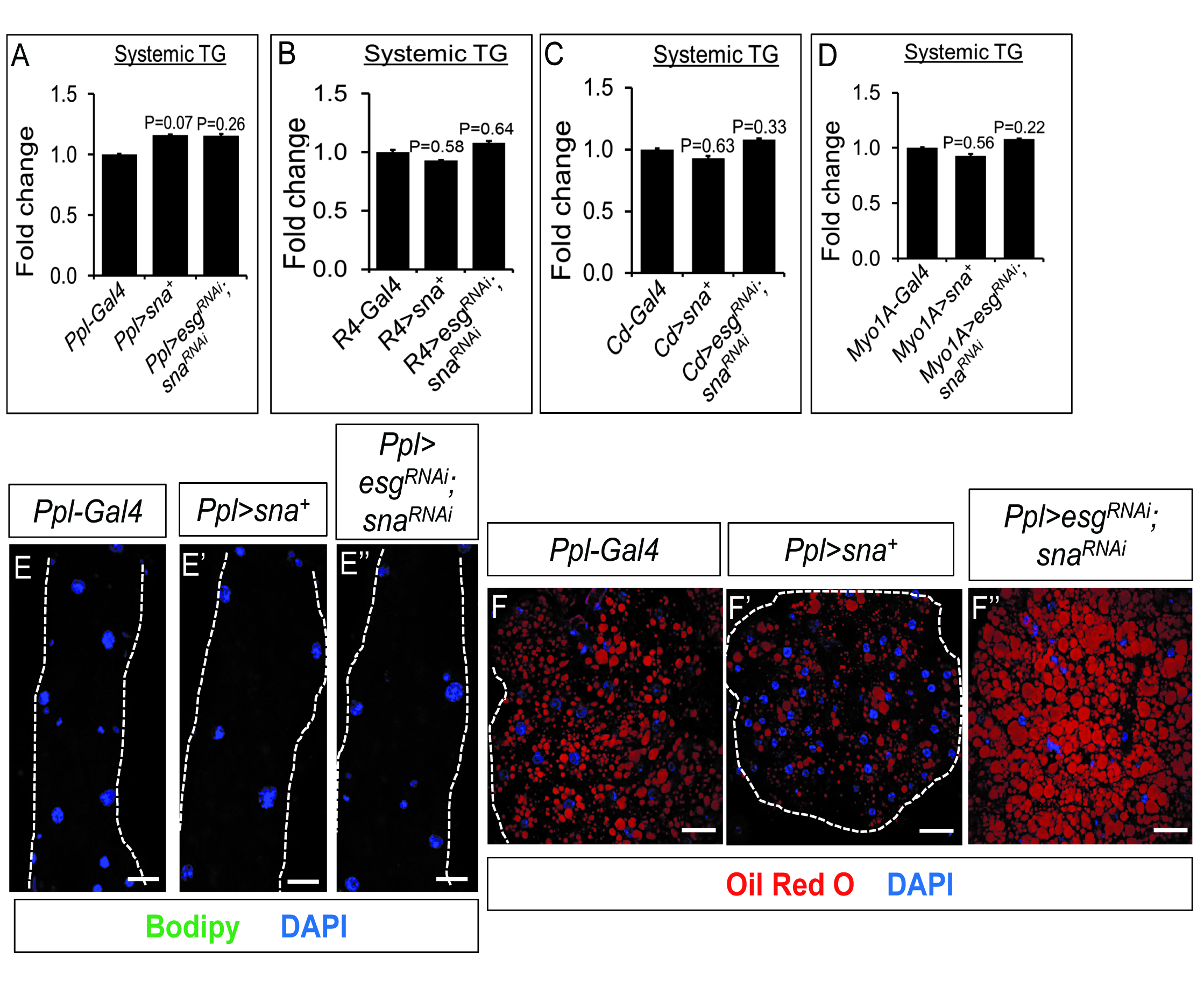

Supplement: S5 Fig — (A-B) Whole-body TG level of 7-day old control flies [Ppl-Gal4 (A) or R4-Gal4 (B)], flies with fat body-specific overexpression of Sna [Ppl>sna+ (A) or R4>sna+ (B)], and flies with fat body- specific knockdown of Sna TFs (Ppl>esgRNAi; snaRNAi (A) or R4> esgRNAi; snaRNAi (B)]. TG levels (μg/μl) were normalized to total protein (μg/μl). Results are the mean ± SEM of 30–40 flies analyzed over at least 3 independent experiments and are expressed as the fold change normalized TG compared with that of the control flies (set to 1.0). (C-D) Whole-body TG level of 7-day old control flies [Cd-Gal4 (C) or Myo1A-Gal4 (D)], flies with intestinal-specific overexpression of Sna [Cd>sna+ (C) or Myo1A>sna+ (D)], and flies with intestinal-specific knockdown of Sna TFs (Cd>esgRNAi; snaRNAi (C) or Myo1A> esgRNAi; snaRNAi (D)]. TG levels (μg/μl) were normalized to total protein (μg/μl). Results are the mean ± SEM of 30–40 flies analyzed over at least 3 independent experiments and are expressed as the fold change normalized TG compared with that of the control flies (set to 1.0). (E-E”) Representative confocal images of hearts stained with Bodipy (green) and DAPI (blue) from 7-day old control flies (Ppl-Gal4) (E), flies with fat body-specific overexpression of Sna (Hand>sna+) (E’), and flies with fat body-specific knockdown of Sna TF genes (Hand>esgRNAi; snaRNAi) (E”). For each genotype, 6 hearts were analyzed. Dotted lines mark the outlines of heart tubes. Scale bar represents 20 μm. (F-F”) Representative confocal images of fat bodies stained with Oil Red O (red) and DAPI (blue) from 7-day old control flies (Ppl-Gal4) (F), flies with fat body-specific overexpression of Sna (Ppl>sna+) (F’), and flies with fat body-specific knockdown of Sna TF genes (Ppl>esgRNAi; snaRNAi) (F”). For each genotype, 6 fat bodies were analyzed. Dotted lines mark the outlines of fat bodies. Scale bar represents 20 μm. (TIF) [file pgen.1008487.s005.tif]

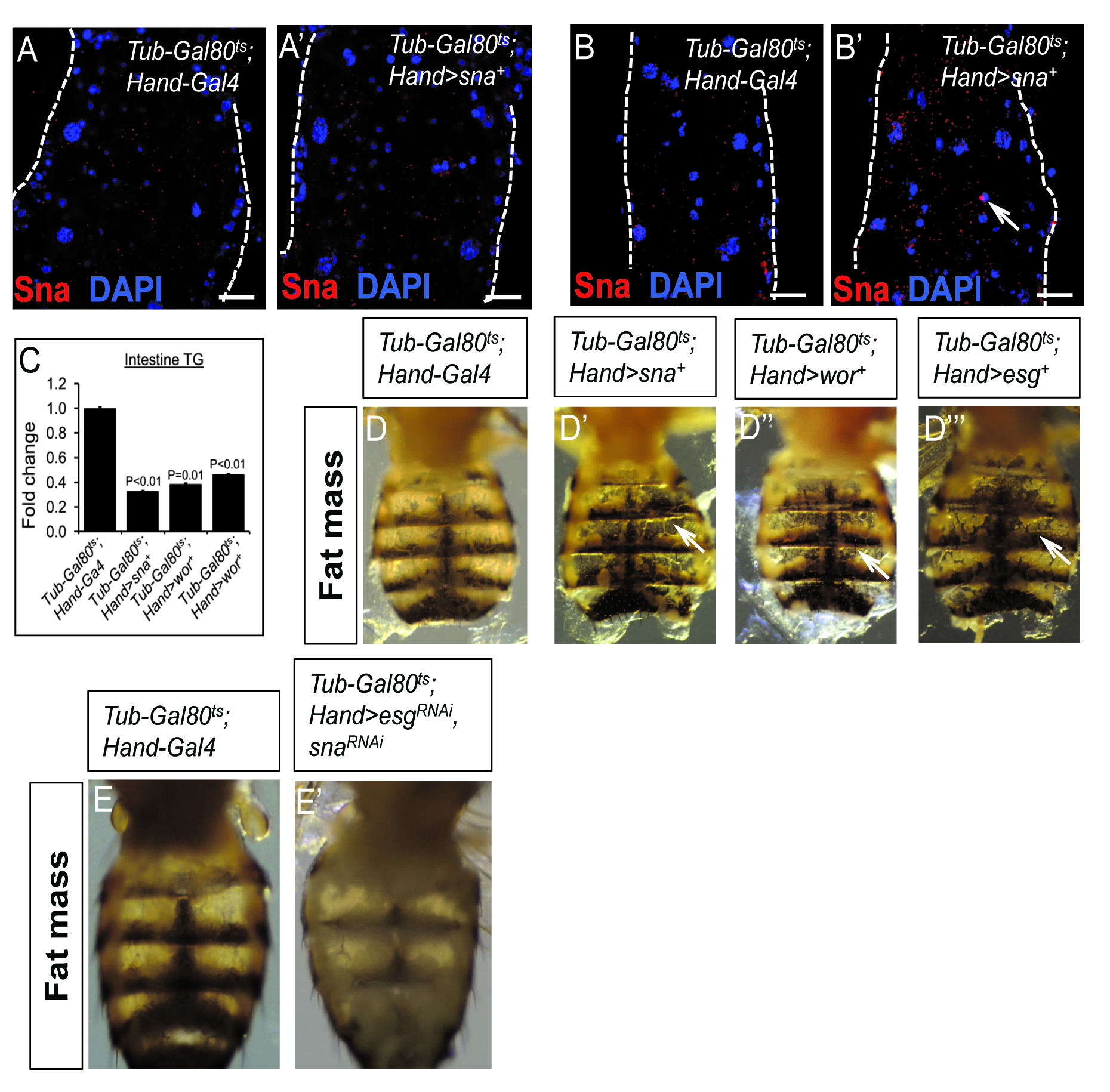

Supplement: S6 Fig — (A-A’) Representative confocal images of hearts immunostained for Sna (red) and co- stained with DAPI (blue) from newly-eclosed control flies (Tub-Gal80ts; Hand-Gal4) or flies bearing Tub-Gal80ts, Hand-Gal4, and UAS-sna+ (Tub-Gal80ts; Hand>sna+) that have been reared from embryo to newly-eclosion at 17°C. For each genotype, 6 hearts were analyzed. Dotted lines mark the outlines of heart tubes. Scale bar represents 20 μm. (B-B’) Representative confocal images of hearts immunostained for Sna (red) and co- stained with DAPI (blue) from 7-day-old control flies (Tub-Gal80ts; Hand-Gal4) and flies bearing Tub-Gal80ts, Hand-Gal4, and UAS-sna+ (Tub-Gal80ts; Hand>sna+) that have been reared from embryo to newly-eclosion at 17°C and from newly-eclosion to 7-day-old adulthood at 29°C. For each genotype, 6 hearts were analyzed. Dotted lines mark the outlines of heart tubes. Arrow denotes Sna localizations to nuclei in the cardiomyocytes. Scale bar represents 20 μm. (C) Intestinal TG level of 7-day old control flies (Tub-Gal80ts; Hand-Gal4) and flies with cardiomyocyte-specific overexpression of Snail TFs in postnatal hearts only (Tub-Gal80ts; Hand>sna+ or Tub-Gal80ts; Hand>wor+ or Tub-Gal80ts; Hand>esg+). TG levels (μg/μl) were normalized to total protein (μg/μl). Results are the mean ± SEM of 30–40 flies analyzed over at least 3 independent experiments and are expressed as the fold change normalized TG compared with that of the control flies (set to 1.0). (D-D”‘) Abdominal fat mass in 7-day old control flies (Tub-Gal80ts; Hand-Gal4) (D) and flies that overexpress Sna TFs in postnatal hearts only (Tub-Gal80ts; Hand>sna+ or Tub-Gal80ts; Hand>wor+ or Tub-Gal80ts; Hand>esg+) (D’-D”‘). White arrows denote gaps in fat mass. (E-E’) Abdominal fat mass in 7-day old control flies (Tub-Gal80ts; Hand-Gal4) (E) and flies with Sna TF gene knockdowns in postnatal hearts only (Tub-Gal80ts; Hand>esgRNAi, snaRNAi) (E’). (TIF) [file pgen.1008487.s006.tif]

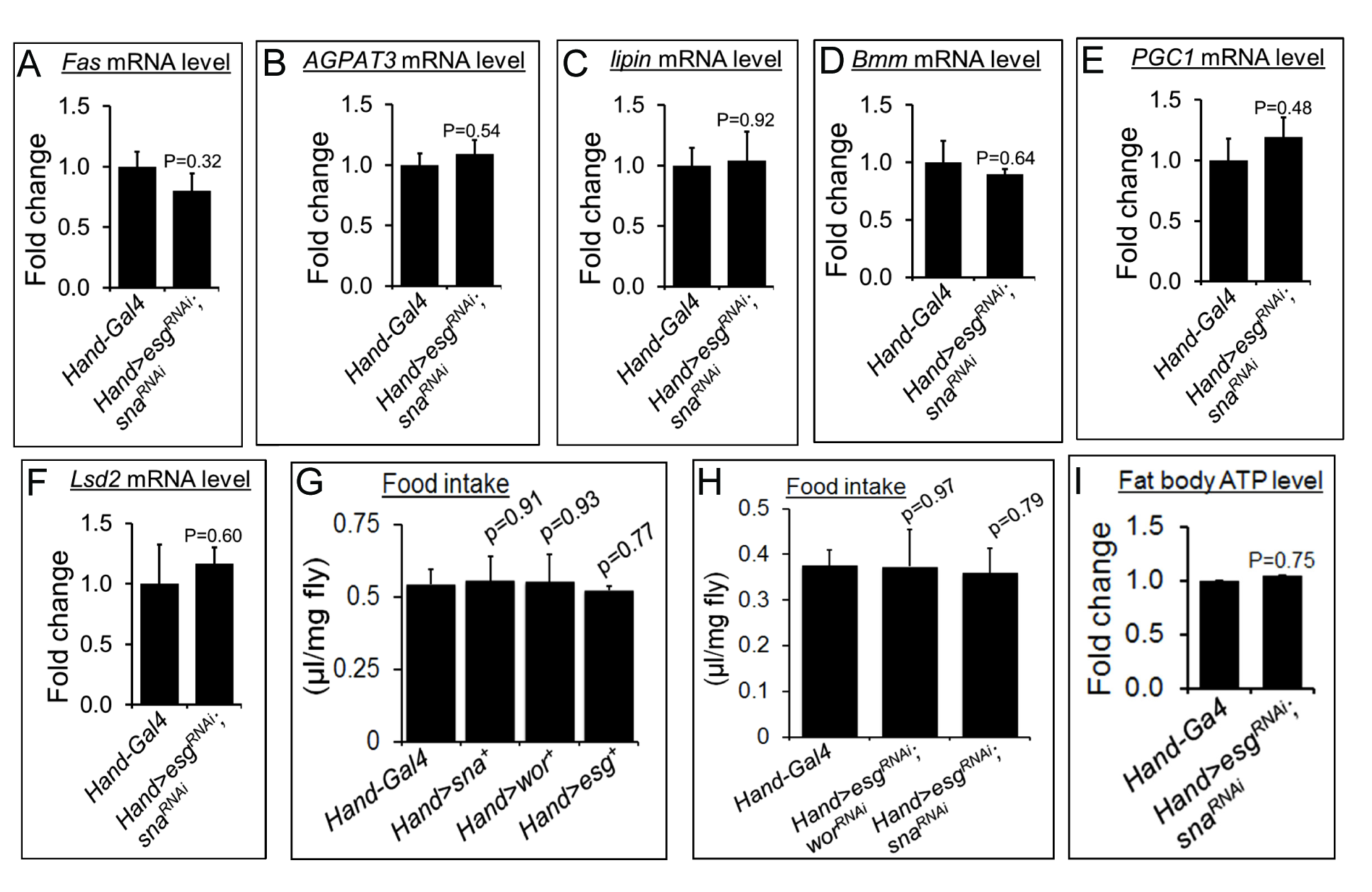

Supplement: S7 Fig — (A-F) Relative mRNA level of FFA synthesis gene Fas (A), TG lipogenesis genes AGPAT3 (B) and Lipin (C), lipolytic gene Bmm (D), mitochondrial biogenesis gene PGC-1/spargel (E), and lipid droplet surface protein gene Lsd2 (F) in hearts of control flies (Hand-Gal4) and flies with cardiomyocyte-specific knockdown of Sna TF genes (Hand>esgRNAi; snaRNAi). Results are the mean ± SEM of 30 hearts analyzed over 3 independent experiments and are expressed as the fold change compared with control hearts (set to 1.0). (G-H) CAFÉ assay of food intake in 1-wk old control flies (Hand-Gal4) and flies with cardiomyocyte-specific overexpression of Sna (Hand>sna+), Wor (Hand>wor+), or Esg (Hand>esg+) (G), or in 1-wk old control flies (Hand-Gal4) and flies with cardiomyocyte-specific inhibition of Sna TFs (Hand>esgRNAi; worRNAi or Hand>esgRNAi; snaRNAi) (H). Results are the mean ± SEM of 30 flies analyzed over at least 3 independent experiments. (I) Steady-state ATP level in the fat bodies of 7-day-old control flies (Hand-Gal4) and flies with cardiomyocyte-specific knockdown of Snail TF genes (Hand>esgRNAi; snaRNAi). Results are the mean ± SEM of fat bodies isolated from 30–40 flies analyzed over 3 independent experiments and are expressed as the fold change compared with control hearts (set to 1.0). (TIF) [file pgen.1008487.s007.tif]

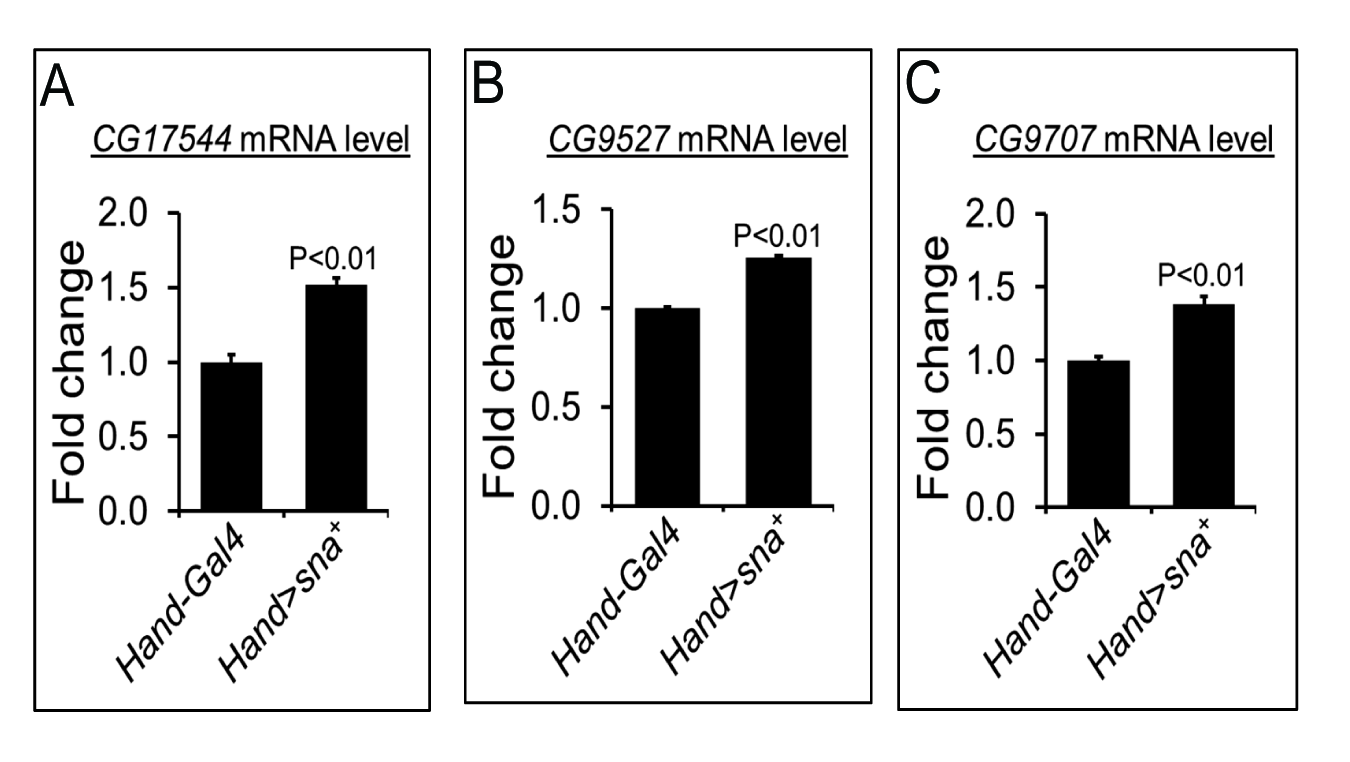

Supplement: S8 Fig — (A-C) Relative mRNA level of genes encoding acyl-coA dehydrogenases (CG17544 and CG9527) (A-B) and gene encoding acyl-coA oxidase 57D-p (CG9707) (C) in fat bodies of 7-day- old control flies (Hand-Gal4) and flies with cardiomyocyte-specific overexpression of Sna (Hand>sna+). Results are the mean ± SEM of 30–40 fat bodies analyzed over 3 independent experiments and are expressed as the fold change compared with control fat bodies (set to 1.0). (TIF) [file pgen.1008487.s008.tif]

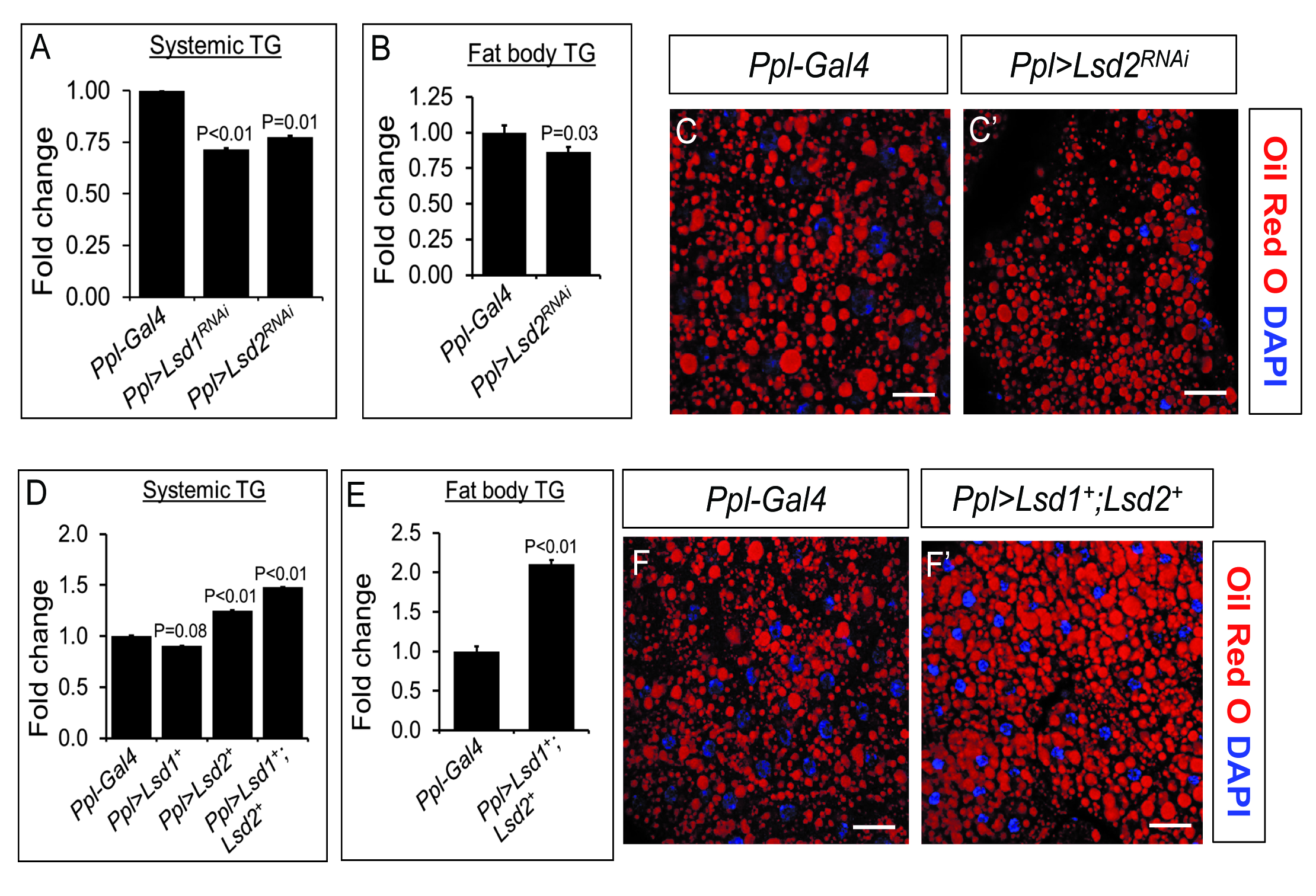

Supplement: S9 Fig — (A-B) Whole-body TG level (A) and fat body TG level (B) of 7-day old control flies (Ppl-Gal4) and flies with fat body-specific inhibition of Lsd1 (Ppl>Lsd1RNAi) or Lsd2 (Ppl>Lsd2RNAi). TG levels (μg/μl) were normalized to total protein (μg/μl). Results are the mean ± SEM of 30–40 flies analyzed over at least 3 independent experiments and are expressed as the fold change normalized TG compared with that of the control flies (set to 1.0). (C-C’) Representative confocal images of abdominal fat bodies stained with Oil Red O (red) and DAPI (blue) from 1-wk-old control flies and flies with Lsd2 knockdown in fat body. For each genotype, at least 6 fat bodies were analyzed. Scale bar represents 20 μm. (D-E) Whole-body TG level (A) and fat body TG level (B) of 7-day old control flies (Ppl-Gal4) and flies with fat body-specific overexpression of Lsd1 (Ppl>Lsd+) or Lsd2 (Ppl>Lsd2+) or both (Ppl>Lsd+;Lsd2+). TG levels (μg/μl) were normalized to total protein (μg/μl). Results are the mean ± SEM of 30–40 flies analyzed over at least 3 independent experiments and are expressed as the fold change normalized TG compared with that of the control flies (set to 1.0). (F-F’) Representative confocal images of abdominal fat bodies stained with Oil Red O (red) and DAPI (blue) from 1-wk-old control flies (F) and flies with Lsd1 and Lsd2 co-overexpression in fat body (Ppl>Lsd+;Lsd2+) (F’). For each genotype, at least 6 fat bodies were analyzed. Scale bar represents 20 μm. (TIF) [file pgen.1008487.s009.tif]

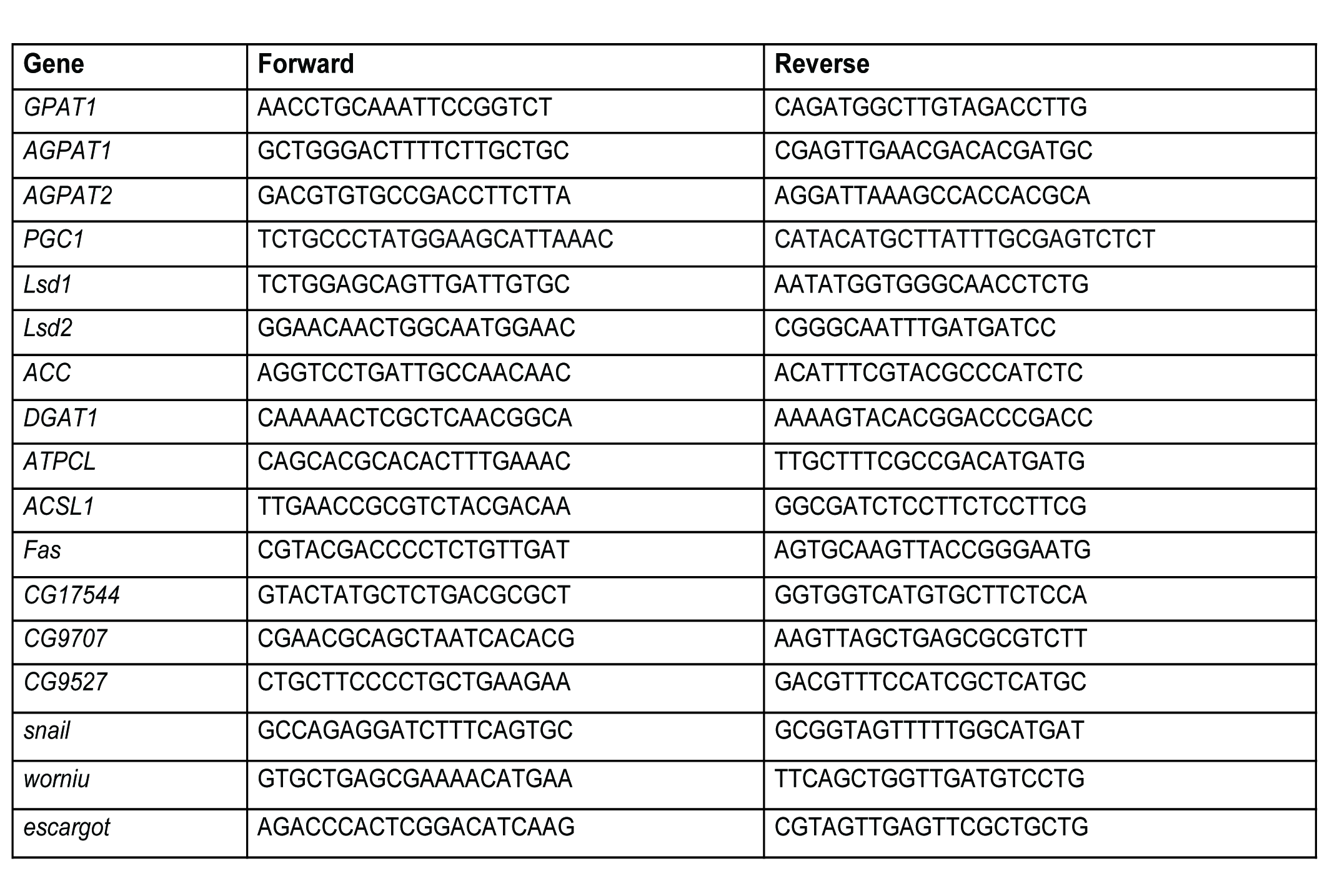

Supplement: S1 Table — (TIF) [file pgen.1008487.s010.tif]
